# Multifunctional and Scalable Nanoparticles for Bimodal Image-Guided Phototherapy in Bladder Cancer Treatment

**DOI:** 10.1007/s40820-025-01717-0

**Published:** 2025-04-18

**Authors:** Menghuan Tang, Sohaib Mahri, Ya-Ping Shiau, Tasneem Mukarrama, Rodolfo Villa, Qiufang Zong, Kelsey Jane Racacho, Yangxiong Li, Yunyoung Lee, Yanyu Huang, Zhaoqing Cong, Jinhwan Kim, Yuanpei Li, Tzu-Yin Lin

**Affiliations:** 1 Department of Biochemistry and Molecular Medicine, UC Davis Comprehensive Cancer Center, University of California Davis, Sacramento, CA 95817 USA; 2 Department of Biomedical Engineering, University of California Davis, Davis, CA 95616 USA; 3 Department of Surgery, School of Medicine, University of California Davis, Sacramento, CA 95817 USA; 4 Division of Hematology/Oncology, Department of Internal Medicine, University of California Davis, Sacramento, CA 95817 USA

**Keywords:** Image-guided phototherapy, Photodynamic and photothermal therapy, Bladder cancer treatment, Photoacoustic imaging, Microfluidics-aided production

## Abstract

Rational design of multifunctional nanoplatforms capable of combining therapeutic effects with real-time monitoring of drug distribution and tumor status is emerging as a promising approach in cancer nanomedicine. Here, we introduce pyropheophorbide a–bisaminoquinoline conjugate lipid nanoparticles (PPBC LNPs) as a bimodal system for image-guided phototherapy in bladder cancer treatment. PPBC LNPs not only demonstrate both powerful photodynamic and photothermal effects upon light activation, but also exhibit potent autophagy blockage, effectively inducing bladder cancer cell death. Furthermore, PPBC LNPs possess remarkable photoacoustic (PA) and fluorescence (FL) imaging capabilities, enabling imaging with high-resolution, deep tissue penetration and high sensitivity for tracking drug biodistribution and phototherapy efficacy. Specifically, PA imaging confirms the efficient accumulation of PPBC LNPs within tumor and predicts therapeutic outcomes of photodynamic therapy, while FL imaging confirms their prolonged retention at the tumor site for up to 6 days. PPBC LNPs significantly suppress bladder tumor growth, with several tumors completely ablated following just two doses of the nanoparticles and laser treatment. Additionally, PPBC LNPs were formulated with lipid-based excipients and assembled using microfluidic technology to enhance biocompatibility, stability, and scalability, showing potential for clinical translation. This versatile nanoparticle represents a promising candidate for further development in bladder cancer therapy.

**Supplementary Information:**

The online version contains supplementary material available at 10.1007/s40820-025-01717-0.

## Highlights

- Pyropheophorbide a–bisaminoquinoline conjugate lipid nanoparticles (PPBC LNPs), formulated with lipid-based excipients and assembled using microfluidics, exhibit excellent biocompatibility and stability, indicating promise for clinical applications.
- PPBC LNPs possess remarkable photoacoustic and fluorescence imaging capabilities, enabling high-resolution, deep tissue penetration and high sensitivity imaging for tracking drug biodistribution and phototherapy efficacy.

**Supplementary Information:**

The online version contains supplementary material available at 10.1007/s40820-025-01717-0.

## Graphical abstract

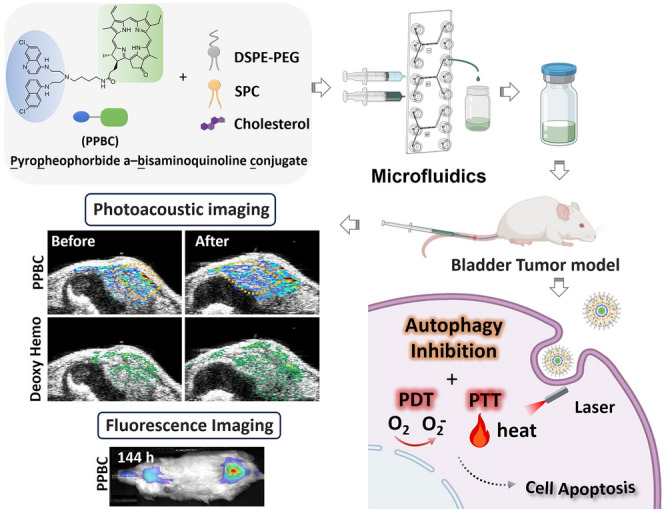

## Introduction

Bladder cancer is a prevalent urological malignancy, with 614,298 new cases and 220,596 deaths reported globally in 2022 [1, 2]. Standard treatments include transurethral resection of bladder tumor (TURBT), intravesical chemotherapy, and immunotherapy for non-muscle invasive bladder cancer (NMIBC), as well as radical cystectomy with chemotherapy for muscle invasive bladder cancer [3, 4]. Despite these interventions, up to 30%–80% of patients experience tumor recurrence within 5 years [5, 6]. Current therapies face several challenges: TURBT or cystectomy alone is usually insufficient to prevent recurrence, while intravesical treatments suffer from poor drug retention, systemic toxicity, and limited penetration into bladder tissue [7, 8]. Chemotherapeutic agents like mitomycin C and Bacillus Calmette–Guérin immunotherapy, although effective, can cause significant side effects and may lead to drug resistance [9].

Photodynamic therapy (PDT) offers a minimally invasive option for bladder cancer treatment [10, 11]. The bladder's accessibility and the feasibility of delivering light through optical fibers make it suitable for PDT. PDT was clinically approved for bladder cancer treatment in 1993 with the photosensitizer Photofrin® [12]. More recently, TLD-1433, a polypyridyl Ru (II) complex, has entered clinical trials for NMIBC treatment [13, 14]. However, PDT suffers from intrinsic limitations, including oxygen dependence, poor selectivity, limited bioavailability, and poor pharmacokinetic properties, all of which constrain its clinical efficacy [10, 15]. Photothermal therapy (PTT) has emerged as an alternative for treating advanced tumors [16, 17]. PTT employs photosensitizers (PSs) to convert laser energy into heat, inducing hyperthermia-mediated cell death. This oxygen-independent mechanism may overcome the hypoxia-related limitations of PDT. Therefore, there is growing interest in developing dual-modal PSs that can elicit both PDT and PTT effects for bladder cancer treatment [18].

PSs typically exhibits near-infrared (NIR) absorption, endowing them with NIR fluorescence (FL) imaging capabilities, which facilitates the discrimination between cancerous and normal tissues at a microscopic scale and supports tumor delineation during intraoperative procedures [19, 20]. While FL imaging shows promise for intraoperative or ex vivo imaging applications due to its high sensitivity, its utility in vivo is limited by relatively low penetration depth and strong optical scattering in biological tissues [21]. In contrast, photoacoustic (PA) imaging, which converts light absorption into acoustic signals, has emerged as a promising tool for in vivo and clinical imaging applications with high-resolution, deep-tissue visualization, overcoming many limitations of conventional optical imaging [22, 23]. Additionally, PA imaging can provide functional biological information, such as blood oxygenation levels, hemoglobin concentration, and metabolic activity, by exploiting differential optical absorption properties of endogenous absorbers like oxy/deoxyhemoglobin [24, 25]. In that context, combining PA imaging with PDT allows real-time, noninvasive monitoring of blood oxygen saturation, a key factor for PDT efficacy, thus enabling optimized treatment planning and improved therapeutic outcomes. However, the field of view (FOV) in PA imaging is highly dependent on the ultrasound (US) transducer, often limiting it to local regions. Furthermore, PA imaging has lower sensitivity compared to FL imaging, which offers a larger FOV and greater sensitivity. Therefore, integrating PA and FL imaging presents a powerful approach for comprehensive cancer diagnostics and treatment monitoring, combining functional information with drug accumulation details, and thereby synergistically overcoming the limitations of each technique.

Nanomedicine holds immense promises for cancer diagnosis and treatment. However, conventional bulk synthesis methods often result in batch-to-batch variations, limiting the clinical translation of nanoparticle-based cancer therapies [26, 27]. To overcome these limitations, microfluidic technology was employed as a superior technique for nanoparticles (NPs) synthesis. Microfluidic systems provide precise control over reaction conditions, allowing for reproducible synthesis of NPs with uniform size and shape [28]. Besides, microfluidics facilitates scalable production, allowing for the large-scale synthesis of NPs without sacrificing quality or consistency [29].

In previous work, we developed a small-molecule self-assembling nanomedicine, pheophorbide a–bisaminoquinoline conjugate (PBC), which exhibited both autophagy inhibition and PDT effects [30]. Thus, PBC ultimately overcomes autophagy-induced resistance in conventional phototherapy, leading to the eradication of oral tumors. However, the clinical translation of PBC NPs has been hindered by several challenges. The presence of stereoisomers in the pheophorbide a ring V may lead to variability in efficacy and safety in vivo, as well as regulatory and manufacturing challenges. In addition, the bulk synthesis method employed is associated with variability in particle sizes and scalability challenges.

To address these limitations and further explore the potential theranostic applications of this nanomedicine in bladder cancer treatment, we introduce a multifunctional and scalable nanotheranostic agent, pyropheophorbide a–bisaminoquinoline conjugate lipid nanoparticles (PPBC LNPs). To enhance the biocompatibility and scalability of PPBC, we first selected pyropheophorbide a (PPA) as the photosensitizing agent to synthesize PPBC, which eliminates the isomer issue. Secondly, we employed microfluidics to synthesize NPs in a stable and scalable manner. After optimizations, we formulated ideal PPBC LNPs with a size of 107 nm, and the lyophilized PPBC LNPs demonstrated excellent long-term stability for up to six months. PPBC LNPs exhibited excellent both PDT and PTT properties as well as autophagy inhibition, which together led to significant cytotoxicity against bladder cancer cells. Moreover, the porphyrin-based structure of PPBC endowed the LNPs with PA/FL bimodal imaging capabilities, where PA imaging enables functional parameter detection, while FL imaging provides high sensitivity for precise phototherapy guidance. This dual imaging approach optimized phototreatment timing, facilitated real-time biodistribution tracking, and enhanced therapeutic precision. By integrating bimodal imaging with phototherapy, PPBC LNPs effectively suppressed tumor growth and, notably, achieved complete tumor eradication in both subcutaneous and orthotopic bladder cancer models, even at low therapeutic doses.

## Experimental Section

### Materials

Pyropheophorbide a–bisaminoquinoline conjugate (PPBC) was synthesized by Frontier Specialty Chemicals Inc. (UT, USA). Soy phosphatidylcholine (SPC) and DSPE-PEG2000 were purchased from Lipoid GmbH (Germany), Cholesterol-NF was purchased from Spectrum chemical (CA, USA). Water for injection, USP, was purchased from Cytiva/HyClone, Ethanol 200 Proof (100%), USP from Fisher Scientific Company (Ontario, Canada). Sucrose EMPROVE® ESSENTIAL, Ph. Eur. NF was purchased from Sigma-Aldrich (MO, USA). Unless otherwise stated, all other chemicals were purchased from Sigma-Aldrich (MO, USA).

PPBC was confirmed by NMR spectra: ^1^H NMR (600 MHz, MeOD) δ 8.99 (s, 1H), 8.89 (s, 1H), 8.51 (s, 1H), 7.76 (t, *J* = 6.6 Hz, 4H), 7.67 (s, 2H), 7.17 (s, 2H), 6.87 (d, *J* = 8.8 Hz, 2H), 6.41 (d, *J* = 8.8 Hz, 2H), 6.12 (d, *J* = 17.9 Hz, 1H), 6.06 (d, *J* = 11.5 Hz, 1H), 5.72 (d, *J* = 5.7 Hz, 2H), 4.48 (d, *J* = 7.3 Hz, 1H), 4.12 (s, 1H), 3.84 (s, 3H), 3.31 (s, 3H), 3.24 (s, 3H), 2.91 (s, 1H), 2.88 (s, 3H), 2.78 (t, *J* = 16.0 Hz, 2H), 2.71 (s, 4H), 2.59 (d, *J* = 4.8 Hz, 1H), 2.51–2.33 (m, 7H), 2.24 (d, *J* = 5.4 Hz, 2H), 2.18–2.09 (m, 1H), 1.79 (d, *J* = 7.3 Hz, 3H), 1.51 (s, 1H), 1.47 (t, *J* = 7.6 Hz, 3H), 1.24 (d, *J* = 19.5 Hz, 3H), 1.15 (d, *J* = 5.3 Hz, 4H).

### Synthesis and Optimization of Nanoformulations

Briefly, PPBC, SPC, DSPE-PEG2000, and cholesterol were dissolved in ethanol and loaded into a syringe (organic phase). A second syringe was filled with water for injection (aqueous phase). Both syringes were connected to a microfluidic chip (Fluidic 187, microfluidic ChipShop, Germany) and mounted onto separate syringe pumps. The nanoformulation was optimized by varying the flow rate ratios of the aqueous phase to the PPBC mixture (organic phase) and adjusting the total flow rate. The organic solvent was removed using TFF to obtain PPBC LNPs. PPBC LNPs was then lyophilized using Labconco FreeZone Plus 6 Liter Freeze Drier (MO, USA) connected to Labconco Stoppering Tray Dryer (MO, USA).

### Characterization of PPBC LNPs

UV–Vis spectrum was obtained with a UV–Vis spectrometer (UV-1800, Shimadzu, Japan). Fluorescence spectrum was collected by a fluorescence spectrometer (RF-6000, Shimadzu, Japan) using the excitation wavelength of 412 nm. Size distribution, polydispersity index (PDI), and zeta potential were measured by dynamic light scattering (DLS) (Malvern, Nano-ZS, UK). The morphology of nanomaterials was observed by a Talos L120C TEM (Thermo Fisher, USA) at an accelerating voltage of 80 kV. The concentration of PPBC was determined by HPLC (Shimadzu LC-2030C 3D plus) at 327 nm using gradient mode (ACN 0.1%TFA and water 0.1%TFA 60% to 100%). The encapsulation efficiency was determined by centrifugal ultrafiltration method. Briefly, PPBC LNPs are centrifuged using a centrifugal filter (10 kDa MWCO, Sartorius, USA) at 15,000 rpm for 15 min. The PPBC concentration is then measured in both the filtrate (bottom chamber) and the upper chamber (retentate) using HPLC. The encapsulation efficiency (EE) was calculated as follows:

EE % = Amount of drug in the retentate/Amount of total drug × 100%

Photothermal stability and photothermal conversion efficiency were assessed by recording the temperatures of PPBC LNPs solutions during heating–cooling cycles. PPBC LNPs solution were placed in 0.5 mL microcentrifuge cap and irradiated with a 680 nm laser. A FLIR thermal camera (C5, Teledyne FLIR, USA, accuracy ± 0.1 °C) was used to record temperature changes over time, and data from heating–cooling cycles were extracted and plotted against time. Various concentrations of PPBC LNPs were prepared by diluting a 1 mM stock solution in 0.9% saline immediately prior to measurements. The photothermal conversion efficiency (*η*) was calculated according to Eq. (1) reported in the literature [31, 32]. A laser power of 0.4 W cm^−2^ at 680 nm and a PPBC LNPs solution of 0.2 mM were selected for these calculations. A concentration of 0.2 mM PPBC LNPs was selected as it falls within the linear range of absorbance at 680 nm (Fig. S3c).

1 $$\eta = \frac{{hs\Delta T_{{\max }} ~ - ~Q_{{{\text{Dis}}}} }}{{I\left( {1 - 10^{{ - A680}} } \right)}}$$

$\eta$ = photothermal conversion efficiency. $\Delta T_{{\max }} = ~T_{{{\text{Max}}}} - ~T_{{{\text{Surr}}}}$ is the temperature difference of PPBC LNPs suspension between the maximum steady-state temperature and the ambient temperature. For 0.2 mM, PPBC LNPs $\Delta T_{{\max }} = 28.9\,^\circ \text{C}$.

Q_*Dis*_ represents heat dissipated from the laser mediated by the solvent and container. *I* is the laser power (0.4 W cm^−2^), and A is the absorbance at 680 nm (A_680_ = 0.959, from Fig. S3c).

*h* is the heat transfer coefficient; *s* is the surface area of the irradiated solution. $hs$ can be obtained from Eq. (2):

2 $$hs = \frac{{mC_{{{\text{water}}}} }}{{\tau s}}$$

*where m* is the mass of the PPBC LNPs solution (ca. 0.0804 mg, 80 μL solution), C_*water*_ is the specific heat capacity of the solvent (water) (C_*water*_ = 4.2 J g^−1^ °C^−1^), and *τs* is the associated time constant. *τs* is obtained from the linear fitting of Eq. (3) in Fig. S3e (τ*s* = 69.81):

3 $$t = - \tau s\,{\text{In}}(\theta )$$

Likewise, Q_*Dis*_ is obtained from Eq. (4)

4 $$Q_{Dis}~ = h_{s} s\Delta T_{{\max ~}}$$

where $h_{s}$ s is determined the same way as above from cooling period of the pure saline solution (τ*s* = 112.5 from Fig. S3f), $\Delta T_{{\max }} = 7.8\,^\circ {\text{C}}$.

From all the above, the photothermal conversion efficiency of PPBC LNPs at 680 nm is $~\eta = 32.7\%$

### ROS Production and Photo-Induced Hyperthermia of PPBC LNPs In Vitro

Singlet oxygen (^1^O_2_) was determined using Singlet Oxygen Sensor Green (SOSG, Thermo Fisher Scientific, USA) as an indicator. SOSG working solution and PPBC LNPs mix solutions were irradiated for 60 s using a 633 nm by LED array (30 mW cm^−2^, Omnilux new-U, PhotoTherapeutics, USA), and then, fluorescence intensity (*Ex*/*Em* = 490/530 nm) was determined by microplate reader (SpectraMax iD5, Molecular Devices, USA).

A drop of an aqueous suspension of PPBC LNPs (50 μM) was placed on a plate and irradiated with 0.6 W cm^−2^ laser (680 nm, Shanghai Xilong Optoelectronics Technology, China) for 3 min. The temperature of the NPs suspension was recorded by a FLIR thermal camera (C5, Teledyne FLIR, USA).

### Cell Culture

T24, Luciferase and GFP-expressing human bladder cancer cells T24, human lung fibroblast cells IMR-90 were cultured in RPMI 1640 supplemented with 10% FBS and 1% penicillin/streptomycin (P/S). Mouse bladder cancer cells UPPL were cultured in DMEM supplemented with 10% FBS and 1% P/S. All cells incubated at 37 °C in a fully humidified atmosphere of 5% CO_2_ in air.

### Cell Viability Assay

Briefly, cells were seeded in 96-well plate (3000 cells/well) and incubated overnight. Cells were then treated with PPBC LNPs and washed after 24 h incubation. For the light-treated groups, cells were irradiated with 633 nm LED array and further incubated for 24 h in parallel with non-light-treated group. Cell viability was quantified using the CellTiter-Glo assay (Promega, USA) and the luminescence intensity was measured by the microplate reader (SpectraMax iD5, USA).

### Cell Uptake Assay

T24 and UPPL were incubated in 96 well glass-bottom plates (3000 cells/well) and then exposed to 0.5 µM of PPBC LNPs for 2, 4, 8, or 24 h. At the specified timepoint, cells were washed and imaged by APX100 microscope (Olympus, Japan). PPBC LNPs was imaged using Cy5 channel, and nuclei was stained by Hoechst 33342 (Invitrogen, USA). In parallel, the uptake of PPBC LNPs by T24 cells was assessed over time (2, 4, 8, and 24 h at 5 µM) and across a range of concentrations (1.25 to 10 µM for 3 h). At each endpoint, T24 cells were washed with PBS and lysed with 0.5% Triton in DMSO. The fluorescence of PPBC was quantified at *Ex*/*Em* = 412/675 nm using the microplate reader. The effect of temperature was assessed by exposing cells to 5 µM of PPBC LNPs at 4 °C for 1 h. To study the uptake pathway, cells were treated with different uptake inhibitors, chlorpromazine (20 µg mL^−1^), genistein (15 µg mL^−1^), or amiloride (20 µg mL^−1^) for 1 h. Without removing the inhibitors, cells were exposed to 5 µM PPBC LNPs for 3 h. The cells were then washed by PBS then lysed, and the fluorescence intensity was measured.

### Colocalization Assay

1 × 10^4^ T24 cells were cultured per well in a 4 chambers cell dish. Next day, cells were treated with 0.5 µM of PPBC LNPs for 2 or 8 h. After washing, cells were stained with LysoTracker™ green (Invitrogen, USA) for 30 min and Hoechst 33342 for 15 min. Cells were visualized using a confocal laser scanning microscopy (CLSM; Carl Zeiss, Germany). Signals of PPBC LNPs were observed under Cy5 channel, and LysoTracker green and Hoechst 33342 were observed under Alexa Fluor 488 and Hoechst 33342 channel, respectively. Pearson’s correlation coefficient was calculated using imaging J.

### Lysosomal Membrane Permeabilization (LMP)

Cells were incubated in 96-well plate (3000 cells/well) and loaded with 100 µg mL^−1^10 kDa Dextran Alexa Fluor™ 488 (Invitrogen, USA) overnight. Next day, cells were washed by PBS, incubated in fresh medium for 2 h and then exposed to chloroquine (10 µM), or PPBC LNPs (10 µM) for 24 h. Cells were then washed with PBS, and nuclei were stained by Hoechst 33342. Images were acquired using APX100 microscope.

### Western Blot Analysis

Cells treated as indicated were collected and lysed using RIPA lysis buffer (Thermo Scientific, USA), followed by protein quantification using a BCA kit (Thermo Scientific, USA). Proteins were separated on a 12% SDS-PAGE gel and transferred onto 0.45 µm polyvinylidene difluoride membranes. After blocking with 2.5% BSA, the membranes were incubated overnight at 4 °C with primary antibodies (1:1000). Membranes were then washed with tris-buffered saline with Tween (TBST) buffer and incubated with secondary peroxidase-conjugated antibodies (1:4000) for 1 h at room temperature, followed by additional washes with TBST. Protein bands were visualized using the ProtoGlow ECL chemiluminescent kit (National Diagnostics, USA) and digitized using a ChemiDoc MP imager (Bio-Rad, USA). The following antibodies were utilized: SQSTM1/p62 (#39749; Cell Signaling Technology), LC3B (#2775; Cell Signaling Technology), Histone H2A.X (#10856–1-AP; Protein Tech), PARP (#9542; Cell Signaling Technology), GAPDH (#60004–1-lg; Protein Tech), HRP-Anti Rabbit (#70745; Cell Signaling Technology), HRP-Anti Mouse (#NC9491974; Fisher Scientific).

### Caspase 3/7 Activity

T24 and UPPL cells were seeded and treated with PPBC LNPs at the specified concentration for 24 h. The cells were then washed with PBS and replaced with fresh medium. The light-treated group was exposed to a 633 nm LED array for 30 s and then incubated for an additional 24 h. Apoptosis activity was measured using the Amplite Fluorometric Caspase 3/7 Assay Kit (AAT Bioquest Inc., USA) according to the manufacturer's instructions.

### Mitochondrial Membrane Potential Analysis

T24 and UPPL cells were seeded at 3000 cells/well and incubated overnight. Cells were then exposed to 0.5 or 1 µM PPBC LNPs for 24 h. Fresh medium was added, and cells were irradiated with 633 nm LED light for 30 s and then incubated for 6 h. Mitochondria were stained with 100 nM tetramethylrhodamine, ethyl ester, perchlorate (TMRE, Invitrogen, USA) for 20 min along with Hoechst 33342 for nuclei staining. After staining, cells were washed with PBS and imaged under APX100 microscope.

### Cellular ROS and MitoROS Detection

UPPL and T24 cells (5.0 × 10^5^ cells/well) were seeded in 6-well plates and incubated overnight. Cells were treated with 0.5 µM PPBC LNPs for 24 h. Following treatment, cells were washed to remove free PPBC LNPs and incubated with CM-H2DCFDA (Invitrogen, USA) and MitoROS™ 580 (AAT Bioquest, USA) for 30 min. Cells were then exposed to 633 nm LED array for 30 s. Immediately after light exposure, cells were collected and analyzed by flow cytometry (Guava easyCyte Flow Cytometer, MilliporeSigma, USA). Data analysis was performed using FlowJo software.

### Cellular Ca^2+^ Influx Detection

UPPL and T24 cells (1.0 × 10^4^ cells/well) were seeded in 96-well plate and incubated overnight. Cells were then exposed to different concentrations of PPBC LNPs for 24 h. Then, cells were washed and exposed to light for 30 s. After 1 h incubation, cells were treated with Rhod-2 AM (Invitrogen, USA) and Fluo-4 AM (AAT Bioquest, USA) for 30 min; then, cells were washed, and the fluorescence intensities were measured by the microplate reader.

### Apoptosis Study

The examination used Annexin V-FITC (BioLegend, CA, USA) and Zombie Red (BioLegend, CA, USA) for the percentage of apoptotic and necrosis cell detection. T24 and UPPL cells were seeded in 6-well plates at a density of 5.0 × 10^5^ cells/well and incubated with 0.2 μM PPBC LNPs for 24 h. Next day, cells were washed and then exposed to the light for 30 s or 5 min. After additional incubation for 6 or 24 h, the cells were harvested and stained with FITC-Annexin V and Zombie red and then analyzed by flow cytometry. Data analysis was accomplished using FlowJo software.

### Animal Model

All animal experiments were conducted in compliance with guidelines and animal protocol (#23784) approved by the Institutional Animal Care and Use Committee at the University of California, Davis. C57BL/6 albino mice (female, 6 weeks old) were purchased from Envigo (Indianapolis, IN, USA). The subcutaneous bladder cancer mouse model was established by inoculating 100 µL of PBS (25% Matrigel) containing 1 × 10^6^ UPPL cells into both flanks of C57BL/6 albino mice. The orthotopic bladder cancer mouse model was created by surgically injecting 15 µL of RPMI-1640 medium containing 1 × 10^6^ GFP and luciferase-expressing T24 cells into the bladder walls of female NRG mice (NOD.Cg-Rag1tm1Mom Il2rgtm1Wjl/SzJ, the Jackson Laboratory). After 3 days, tumor establishment was confirmed by bioluminescence imaging. Animals received post-surgery pain management for 3 days.

### In Vivo/Ex Vivo Near-Infrared (NIR) Fluorescence Imaging

C57BL/6 albino and NRG mice bearing subcutaneous or orthotopic bladder tumors were intravenously (i.v.) injected with PPBC LNPs (10 mg kg^−1^). Tumor progression was monitored by bioluminescence imaging. D-luciferin (150 µL, 20 mg mL^−1^) was injected into mice, and imaging was performed using the Lago X (Spectral Instruments Imaging, USA). Biodistribution of PPBC LNPs was also imaged by Lago X (*Ex*/*Em* = 675/730 nm) at specified intervals. After 24 h of injection, mice were killed, and their main organs and bladder were harvested for ex vivo imaging.

### Ultrasound and Photoacoustic (US/PA) Imaging

All US/PA images were acquired using the Vevo F2/LAZR-X system (FujiFilm VisualSonics, Inc., Canada) equipped with a 256-element linear array US transducer (UHF57x, FujiFilm VisualSonics, Inc.), operating at a frequency range of 25–57 MHz, and a nanosecond pulsed laser (5 ns, 10 Hz) tunable between 680 and 970 nm at 5 nm intervals. Images were initially processed using VevoLAB software (version 5.8.2) and further analyzed using a custom image processing algorithm developed in MATLAB (R2024a). For imaging of PPBC LNPs in solution, a tube phantom (0.3 mm ID, 0.6 mm OD, Instech polyurethane (PU) tubing) containing 50 µL of PPBC LNPs (1 mg mL^−1^) in deionized (DI) water was utilized. The 3D US/PA imaging was performed at 680 nm over a 10 mm range with 0.15 mm step intervals. For in vitro imaging of cells treated with PPBC LNPs, a gelatin-based, tissue-mimicking dome-shaped phantom was prepared. Briefly, T24 bladder cancer cells were incubated with PPBC LNPs (2 and 10 µM) for 24 h, followed by trypsinization. The cells were counted to a concentration of 2000 cells µL^−1^ and subsequently mixed with an equal volume of 16% gelatin. This mixture was cast onto an 8% gelatin phantom base containing 0.2% silica, forming dome-shaped inclusions (~ 40 µL). PA images were acquired by sweeping wavelengths from 680 to 970 nm in 5 nm steps. The spectral PA signals were processed in MATLAB based on the PPBC LNPs spectrum obtained from tube phantom imaging and overlaid with the US images. For in vivo US/PA imaging, mice bearing orthotopic bladder tumors were administered PPBC LNPs (i.v. 10 mg kg^−1^) and imaged at various timepoints: 0 h (pre-injection), 6 h, 24 h before and after laser irradiation (0.2 W cm^−2^, 1.5 min), and 48 h. PA images were acquired by sweeping from 680 to 970 nm in 5 nm steps. The acquired PA signals were unmixed based on the known extinction coefficients of deoxyhemoglobin, oxyhemoglobin, and the absorption spectrum of PPBC LNPs. Each processing cycle generated B-mode images with overlaid signals for PPBC LNPs, deoxyhemoglobin, oxyhemoglobin, oxygen saturation, and total hemoglobin concentration. For quantitative analysis, PA images with different colormaps were converted to greyscale, and the total signal was quantified by counting the pixel intensity within the tumor region defined by the B-mode images. The total signal was normalized to the tumor area for comparative analysis.

### In Vivo Antitumor Studies

For subcutaneous bladder tumor models, mice were randomly divided into two groups when tumors reached approximately 100 mm^3^. The groups were i.v. administered water for injection or PPBC LNPs (10 mg kg^−1^) once per week for two weeks. Laser treatment was applied 24 h post-injection (0.6 W cm^−2^, 3 min). At the end of the treatment, tumors and main organs were collected for further histological analysis. For orthotopic bladder tumor models, mice were randomized into two groups and i.v. administered water for injection or PPBC LNPs (10 mg kg^−1^). Laser treatment (0.2 W cm^−2^, 1.5 min) was applied 24 h after drug administration. Tumor progression was monitored using bioluminescence imaging.

### Immunohistochemistry

Slides were dewaxed, and antigen retrieval was performed for 45 min with citrate buffer at pH 6.0 in a Decloaking Chamber (Biocare Medical, USA) at 125 °C and 15 min. Slides were blocked with normal goat serum then incubated with a rabbit polyclonal antibody Cleave Caspase 3 (#9661, Cell Signaling Technology) with 1:500 dilution, a rabbit monoclonal antibody Ki67 (#12202, Cell Signaling Technology) with 1:1000 dilution, a rabbit polyclonal antibody LC3B (#PA5-32254, Thermo Fisher) with 1:400 dilution, a mouse monoclonal antibody SQSTM1/p62 (#88588, Cell Signaling Technology) with 1:250 dilution. Slides were incubated overnight at room temperature in a humidified chamber, followed by a biotinylated goat anti-rabbit secondary antibody (1:1000, Vector Labs, USA). The Vectastain ABC Kit Elite Kit and a diaminobenzidine Peroxidase Substrate Kit (Vector Labs, USA) were used for amplification and visualization of signal, respectively.

### Statistical Analysis

The experimental data were statistically analyzed using GraphPad Prism 10. Data were reported as the mean ± standard deviations (SD). Data statistics were analyzed by calculating the *t* test between two groups: ns.: not significant; *p < 0.05; **p < 0.01; ***p < 0.001; ****p < 0.0001.

## Results and Discussion

### Formulation and Characterization of the PPBC LNPs

**Fig. 1:**
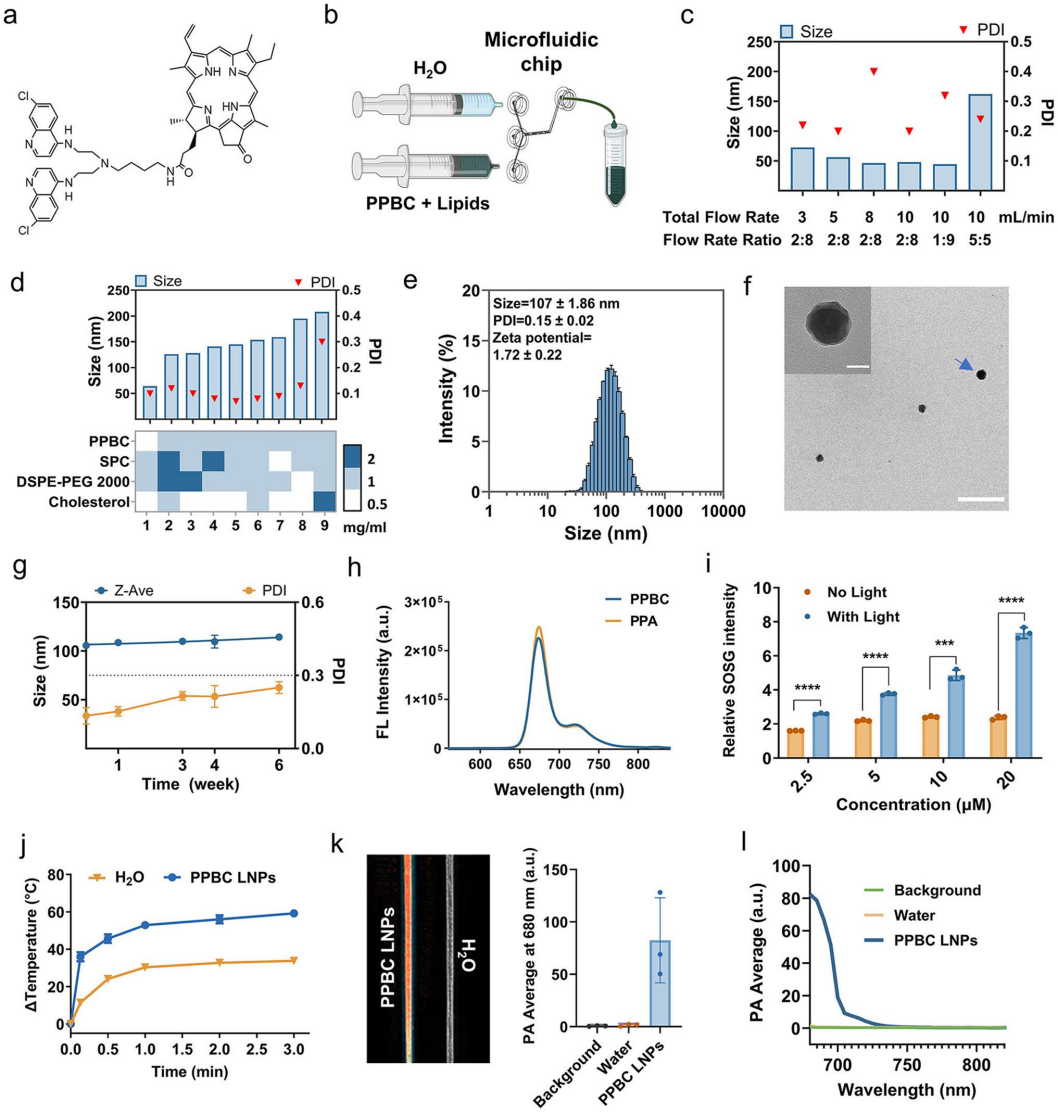
**a** Chemical structure of PPBC. **b** Schematic illustration of the microfluidics system employed to synthesize PPBC LNPs. **c** Optimization of PPBC LNPs size and PDI by varying the TFR, FRR, and **d** the concentration of lipid ingredients ratios (*n* = 3). **e** Representative DLS data and** f** TEM image of reconstituted PPBC LNPs; scale bar: 50 nm (insert), 400 nm. **g** Stability of reconstituted PPBC LNPs at + 4 °C. **h** Fluorescence (FL) spectra (excitation, 412 nm) of PPBC and PPA. **i** Photodynamic effect (^1^O_2_ production) of PPBC LNPs measured by using SOSG as an ROS indicator. **j** Photo-induced hyperthermia of PPBC LNPs (*n* = 3). **k** PA signal generation from PPBC LNPs in aqueous solution. **l** Spectroscopic PA amplitude of PPBC LNPs at 680–820 nm

PPBC monomer was synthesized by conjugating pyropheophorbide and bisaminoquinoline, and its structure was confirmed by NMR spectra (Figs. 1a and S1). SPC, DSPE-PEG2000, and cholesterol were incorporated into the NPs to improve their stability and biocompatibility in vivo. Subsequently, microfluidic platform was utilized to synthesize LNPs, which allows precise control over mixing conditions and enables large-scale production (Figs. 1b and S2a). As shown in Fig. 1c, a, total flow rate (TFR) of 10 mL min^−1^ and a flow rate ratio (FRR) of 2:8 (ethanol/water) produced the most ideal NPs. Under these conditions, PPBC LNPs had an average hydrodynamic diameter of 48.1 nm with a polydispersity index (PDI) of 0.20. Further optimization of component ratios indicated that a formulation of PPBC: SPC: DSPE-PEG2000: Cholesterol at 1:2:2:1 yielded uniform NPs with an average size of 125 nm and the PDI < 0.2 (Figs. 1d and S2b, c).

Lyophilization has been shown to effectively enhance the long-term stability of various NPs [33]. Therefore, to improve their suitability for clinical use, PPBC LNPs were lyophilized in the presence of 8% sucrose as cryoprotectant and 20 mM Tris–HCl buffer to maintain pH at 8.2. Sucrose was selected from the literature as an effective cryoprotectant [34]. Upon lyophilization, PPBC LNPs were easily reconstituted using water for injection (Fig. S2d). The reconstituted LNPs exhibited an average size of 107 nm, a typical spherical morphology, a narrow size distribution with a PDI of 0.15, and a near neutral charge of 1.72 mV (Fig. 1e, f). PPBC demonstrated a high encapsulation efficiency of > 98%. Moreover, the lyophilized PPBC LNPs powder at + 4 °C for 6 months did not affect its stability (Fig. S2e), while reconstituted PPBC LNPs remained stable for at least 6 weeks at + 4 °C (Fig. 1g). These findings highlight the potential of PPBC LNPs for further scale-up and clinical applications.

### Phototheranostic Properties

Given the porphyrin-derived structure of PPBC, PPBC LNPs were investigated for their phototheranostic properties. The fluorescence spectra showed that PPBC exhibited the same characteristic emission wavelength as PPA, indicating that PPBC LNPs maintain the optical properties of PPA (Fig. 1h). To evaluate the phototherapeutic effects of PPBC LNPs, reactive oxygen species (ROS) production and photo-induced hyperthermia were studied in vitro. As shown in Fig. 1i, the fluorescence intensity of the singlet oxygen (^1^O_2_) sensor green (SOSG) increased in a dose-dependent manner under light irradiation, while remaining unchanged in the absence of light, confirming that ROS production by PPBC LNPs is effectively triggered by light. The hyperthermia generated by PPBC LNPs reached 59 °C under light irradiation (Fig. 1j), a temperature sufficient to cause irreversible damage to cancer cells within 2 min [35]. Temperature of PPBC LNPs solution increase correlated with laser power, solution concentration, and exposure time, indicating heat generation tenability. The thermal generation was shown to be linear in the range of 0.05 to 0.4 mM and then showed a saturation effect for higher concentrations (Fig. S3a, b). This linearity was consistent with the PPBC LNPs solution absorbance at 680 nm (Fig. S3c). Moreover, PPBC LNPs exhibited excellent thermal photostability, withstanding several consecutive cycles of heating and cooling without significant loss of heat generation capacity (Fig. S3d). The photoconversion efficiency (*η*) of PPBC LNP was calculated as 32.7% (Fig. S3e, f). These results revealed that PPBC LNPs exhibit both potent PDT and PTT properties, indicating their potential for effective phototherapy. Given the strong optical absorption of PPBC LNPs in NIR region, their potential as a photoacoustic (PA) imaging contrast agent was evaluated (Fig. S2f). The PPBC LNPs solution showed significantly enhanced PA contrast under laser excitation at 680 nm compared to water and background controls (Fig. 1k). In addition, the PA signal spectrum in the NIR range closely aligned with the optical absorption spectrum of PPBC LNPs, demonstrating their effectiveness as a PA contrast agent within the NIR region (Fig. 1l).

### Cellular Uptake and Anti-Lysosomal/Autophagy Effects

**Fig. 2:**
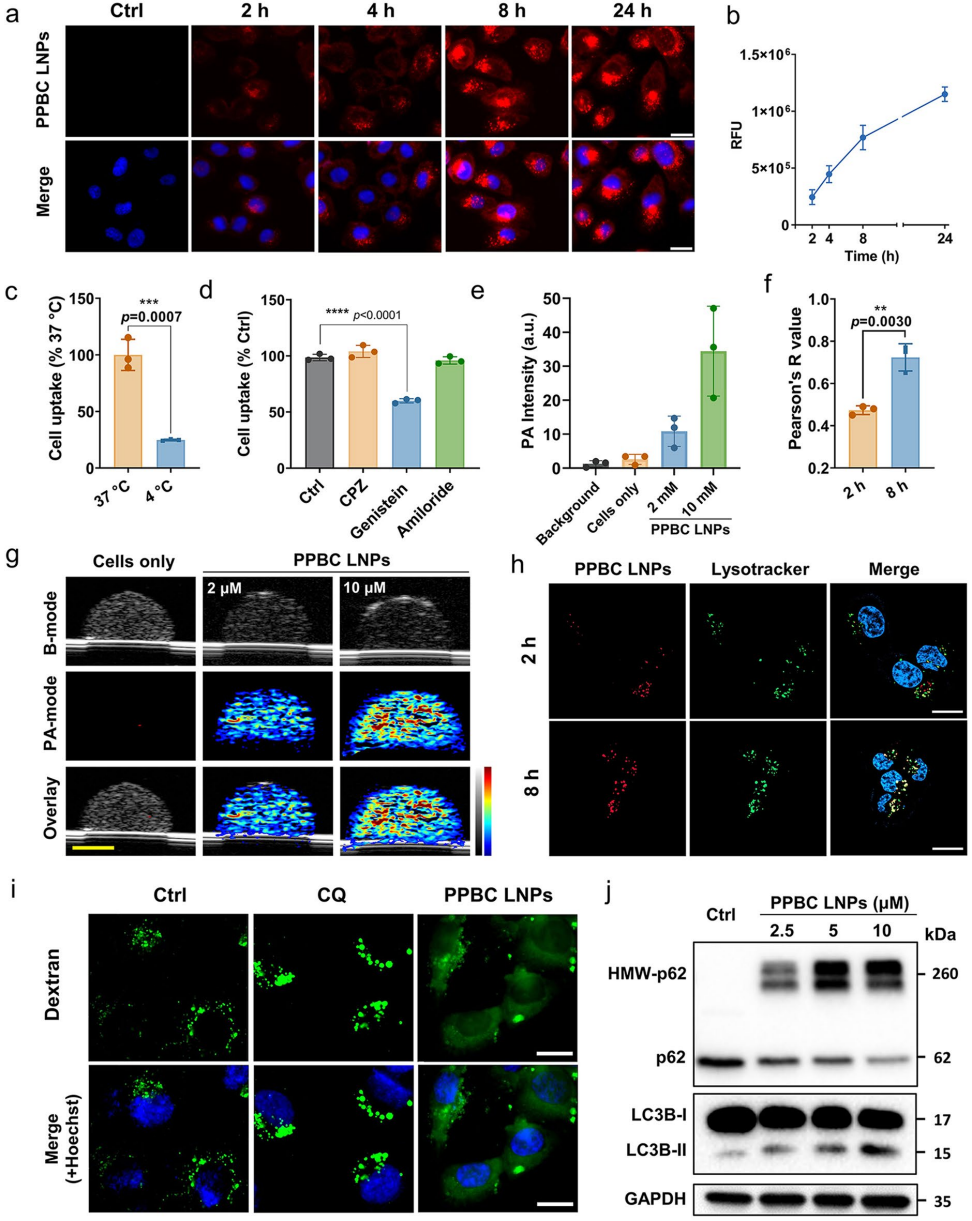
**a** Representative image showing the uptake of PPBC LNPs by T24 bladder cancer cells over time, with increasing signal intensity indicating PPBC LNP uptake; scale bar: 50 μm. **b** Time-dependent and **c** energy-dependent cell uptake of PPBC LNPs. **d** Effect of various endocytosis inhibitors on cellular uptake of PPBC LNPs by T24 cells. The uptake was significantly inhibited by treatment with genistein (20 µM), an inhibitor of caveolae-mediated endocytosis. **e** Corresponding quantification of PA signals shown in **g**. **f** Pearson correlation coefficient (Pearson’s R) for colocalization shown in **h**. **g** US/PA images of the PPBC LNPs-labeled T24 cells at 680 nm; scale bar: 2 mm. **h** Colocalization analysis of PPBC LNPs with lysosomes; cell nuclei were stained with Hoechst 33342; scale bar: 20 μm. **i** Representative images of Dextran-AF 488-loaded T24 cells exposed to chloroquine (CQ,10 μM) or PPBC LNPs (10 μM) for 24 h; cell nuclei were stained with Hoechst 33342; scale bar: 20 μm. **j** Immunoblotting of autophagy-related proteins (p62, LC3B) in T24 cells treated as indicated for 12 h

To evaluate the cellular uptake, T24 human bladder cancer cells were treated with PPBC LNPs, and fluorescence images were acquired at 2, 4, 8, and 24 h post-treatment. As shown in Figs. 2a, b and S4, PPBC LNPs were rapidly internalized by the bladder cancer cells and exhibited a time and dose-dependent accumulation. To further elucidate the cellular uptake mechanism of PPBC LNPs, the effects of various endocytosis inhibitors on their internalization were tested. T24 cells were pretreated with different uptake inhibitors including chlorpromazine (CPZ, clathrin-mediated endocytosis inhibitor), genistein (caveolae-mediated endocytosis inhibitor), and amiloride (macropinocytosis inhibitor). Notably, incubation at low temperature and exposition to genistein significantly inhibited the cellular uptake of PPBC LNPs, while other inhibitors did not show significant effects (Fig. 2c, d). The inhibitory effect of genistein was dose-dependent manner (Fig. S5). This finding suggests that the internalization of PPBC LNPs in T24 cells is an energy-dependent process that primarily occurs through caveolae-mediated endocytosis. Subsequently, the PA signal of cells treated with varying concentrations of PPBC LNPs was monitored. In a tissue-mimicking imaging phantom containing PPBC LNPs-uptake cells, a strong PA signal was clearly observed (Fig. 2g), with the signal intensity increasing in a concentration-dependent manner (Fig. 2e). These results further highlight the potential of PPBC LNPs not only as a photosensitizer for PDT but also as an effective imaging contrast agent for both PA and FL imaging.

Since PPBC was designed as a conjugate of a photosensitizer and an autophagy inhibitor, it was hypothesized that it would accumulate in lysosomes and further lead to autophagy inhibition. To test this, we studied the colocalization of PPBC LNPs with lysosomes using CLSM. As shown in Fig. 2f, h, the signals of PPBC LNPs increasingly overlapped with those of LysoTracker over time, reaching a Pearson correlation coefficient of 0.73 after 8 h of incubation, indicating effective lysosomal accumulation of PPBC LNPs. To further assess the impact of PPBC LNPs on lysosomal integrity, we treated cells with Dextran-AF 488, a fluorescent marker typically confined within intact lysosomes. In both T24 and UPPL cells, PPBC LNPs treatment induced a diffuse cytoplasmic distribution of Dextran-AF 488, suggesting lysosomal membrane permeabilization (LMP) (Figs. 2i and S6). Notably, under the same concentration, chloroquine (CQ), a well-known autophagy inhibitor [36], exhibited weaker LMP compared to PPBC LNPs. We next detected the level of key autophagy markers microtubule-associated protein 1 light chain 3 (LC3B) and Sequestosome 1 (SQSTM1/p62) using western blot analysis. Autophagy inhibition is generally indicated by simultaneously increased levels of LC3B-II and p62 [37]. Compared to control group, cells treated with PPBC LNPs displayed a dose-dependent rise in LC3B-II. Intriguingly, we noticed significant production of high molecular weight (HMW)-p62 species induced by PPBC LNPs, while mono-p62 levels correspondingly decreased (Figs. 2j and S7a, b). Previous studies suggested that redox stress caused by drugs or deficiencies in autophagy-related genes can induce the production of HMW-p62, indicating p62 oligomerization and impaired autophagy [38, 39]. Therefore, the formation of HMW-p62 appears to be attributed to the autophagy inhibition and moderate redox stress induced by PPBC LNPs. This autophagy inhibition feature is a key attribute of PPBC, especially considering that conventional PDT has been shown to activate autophagy, which can contribute to therapeutic resistance [30]. By inhibiting autophagy, PPBC holds substantial potential to overcome this resistance mechanism, thereby enhancing the therapeutic efficacy of the photosensitizer in bladder cancer treatment.

### In Vitro Evaluation of Phototherapeutic Effects

**Fig. 3:**
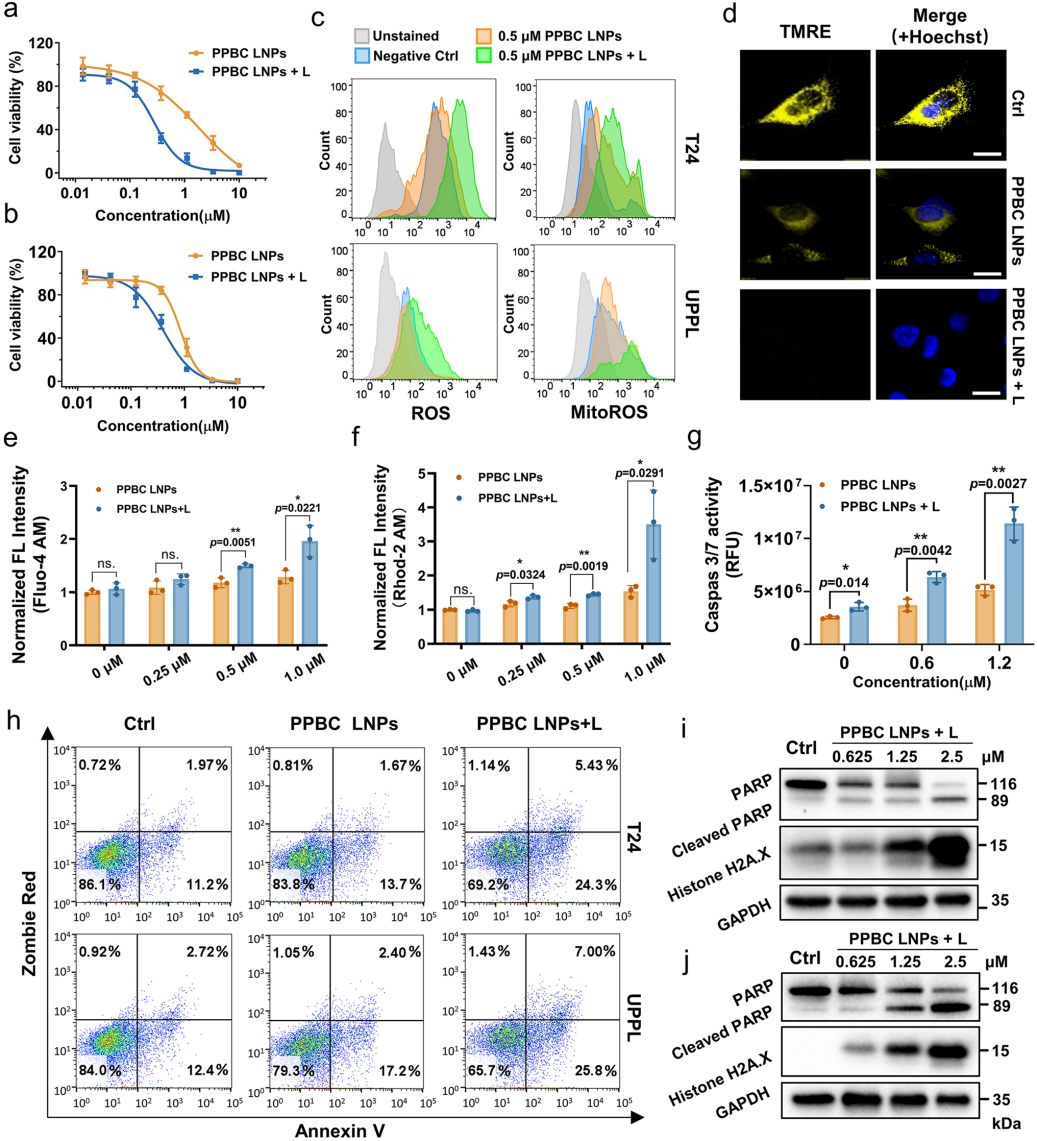
**a** Viability curves of T24 and **b** UPPL bladder cancer cells exposed to PPBC LNPs with or without light treatment (*n* = 3). **c** Flow cytometry analysis of ROS and MitoROS generation in cells treated as indicated. **d** The MMP analysis using TMRE as an indicator; cell nuclei were stained with Hoechst 33342; scale bar: 20 μm. **e** Intracellular and **f** mitochondrial calcium (Ca^2^⁺) influx in T24 cells exposed to PPBC LNPs with or without light treatment. **g** Increased caspase 3/7 activity in T24 cells upon PPBC LNPs (0.5 µM)-mediated phototherapy. **h** Apoptosis assay of bladder cancer cells treated as indicated (0.2 µM). **i** Immunoblotting of apoptosis and DNA damage-related protein (PARP, cleaved-PARP, Histone H2A.X) in T24 and **j** UPPL cells treated with PPBC LNPs plus light. L: with light irradiation, 30 mW cm^−2^ (633 nm LED array) for 30 s

The PPBC LNPs-mediated phototherapy effect was then explored in both human and mouse bladder carcinoma cell lines. As shown in Fig. 3a, b, PPBC LNPs exhibited potent cytotoxicity even in the absence of light, primarily due to their inherent autophagy-blocking property. Importantly, light treatment significantly enhanced the anticancer effects of PPBC LNPs. In T24 cells, the IC_50_ value of light-treated group was 0.28 µM, which was sevenfold lower than that of the non-light-treated group (2.1 µM) (Fig. S8a). Moreover, the cytotoxicity of PPBC LNPs followed a light-dose-dependent trend (Fig. S8b). Notably, PPBC LNPs exhibited lower toxicity toward normal cells (IMR-90), with an IC50 of approximately 25 µM (Fig. S9). It is well known that PDT relies on generating ROS, particularly ^1^O_2_, to induce cancer cell death [40]. To detect intracellular ROS levels, we used 2’,7’-dichlorofluorescein diacetate (DCF-DA) as an indicator. As shown in Fig. 3c, ROS levels were significantly elevated following PPBC LNPs-mediated phototherapy, whereas PPBC LNPs treatment alone showed minimal changes compared to the control. Given that mitochondria are the primary source of ROS in cells, we also measured mitochondrial ROS (MitoROS) level. Consistent with overall ROS production, a significant burst of MitoROS was observed in the light-treated group. PDT-induced oxidative stress can damage mitochondria, leading to mitochondrial dysfunction [41, 42]. One of the hallmarks of mitochondrial dysfunction is depolarization of the mitochondrial membrane, reflected by a reduction or loss of mitochondria membrane potential (MMP) [43]. To evaluate MMP, we used tetramethylrhodamine, ethyl ester (TMRE), a dye that selectively accumulates in the mitochondria due to the negative charge of MMP. Healthy mitochondria with high MMP can attract and retain more TMRE, leading to a strong fluorescent signal. Among all groups, only PPBC LNPs plus light treatment group showed negligible TMRE signals, which indicated a loss of MMP and mitochondria damage in bladder cancer cells (Fig. 3d).

Mitochondria are key organelles that involved in mediating cellular Ca^2+^ homeostasis, which regulating various cell signaling pathways, such as metabolism, cell division, and cell death [44, 45]. Ca^2+^ enters the mitochondria through the mitochondrial calcium uniporter (MCU) driven by the MMP [46]. When MMP is compromised, the MCU tends to become dysregulated, leading to uncontrolled Ca^2+^ influx and overload. Since PPBC LNPs-mediated phototherapy caused MMP depolarization, we subsequently evaluated Ca^2^⁺ influx using Fluo-4 AM to measure intracellular Ca^2^⁺ level and Rhod-2 AM to detect mitochondrial Ca^2^⁺ level. As shown in Fig. 3e, f, compared to control group, PPBC LNPs with light triggered Ca^2+^ overload in both the cytoplasm and mitochondria, while PPBC LNPs-only group did not induce a dramatic change in Ca^2+^ influx. These findings are consistent with previous results, in which PPBC LNPs plus light exhibited enhanced mitochondrial damage, whereas PPBC LNPs alone had a limited impact on mitochondria.

To gain further insight into the PPBC LNPs-mediated cytotoxicity, we evaluated indicators of apoptosis, caspase-3/7 activity. A dose-dependent increase in caspase-3/7 activity was observed in both light-treated and non-light-treated groups, and light treatment significantly triggered a surge in caspase-3/7 activity in T24 cells (Fig. 3g). To further confirm the apoptosis induced by PPBC LNPs-mediated phototherapy, we employed Annexin V/Zombie Red (ZR) staining. Flow cytometry analysis showed that even at a low concentration of 0.2 µM, PPBC LNPs combined with light induced the highest level of apoptosis compared to all other groups in both bladder cancer cell lines (Fig. 3h). For T24 cells, the combined early (Annexin V + /ZR-) and late apoptotic (Annexin V + /ZR +) cell populations were 30%, 15%, and 13% for PPBC plus light, PPBC LNPs-only, and control groups, respectively. At higher doses of PPBC LNPs and light, nearly 80% of the cells exhibited late apoptosis or necrosis (Fig. S10).

We further evaluated the downstream apoptotic marker poly (ADP-ribose) polymerase (PARP), the substrate for caspase-3, and the DNA damage marker Histone H2A.X. Western blot analysis revealed a significant, dose-dependent increase in the levels of cleaved-PARP, and Histone H2A.X following PPBC LNPs-mediated phototherapy, confirming the induction of apoptosis and extensive DNA damage (Figs. 3i, j and S7c, d). These findings further corroborate the apoptosis-inducing effects of PPBC LNPs-mediated phototherapy in bladder cancer cells. Taken together, we concluded that PPBC LNPs-mediated phototherapy caused mitochondria dysfunction and further induced caspase 3/7-dependent apoptosis in bladder cancers, resulting in cell death.

### Dual PA and NIR FL Imaging in Bladder Cancer Xenograft Model

**Fig. 4:**
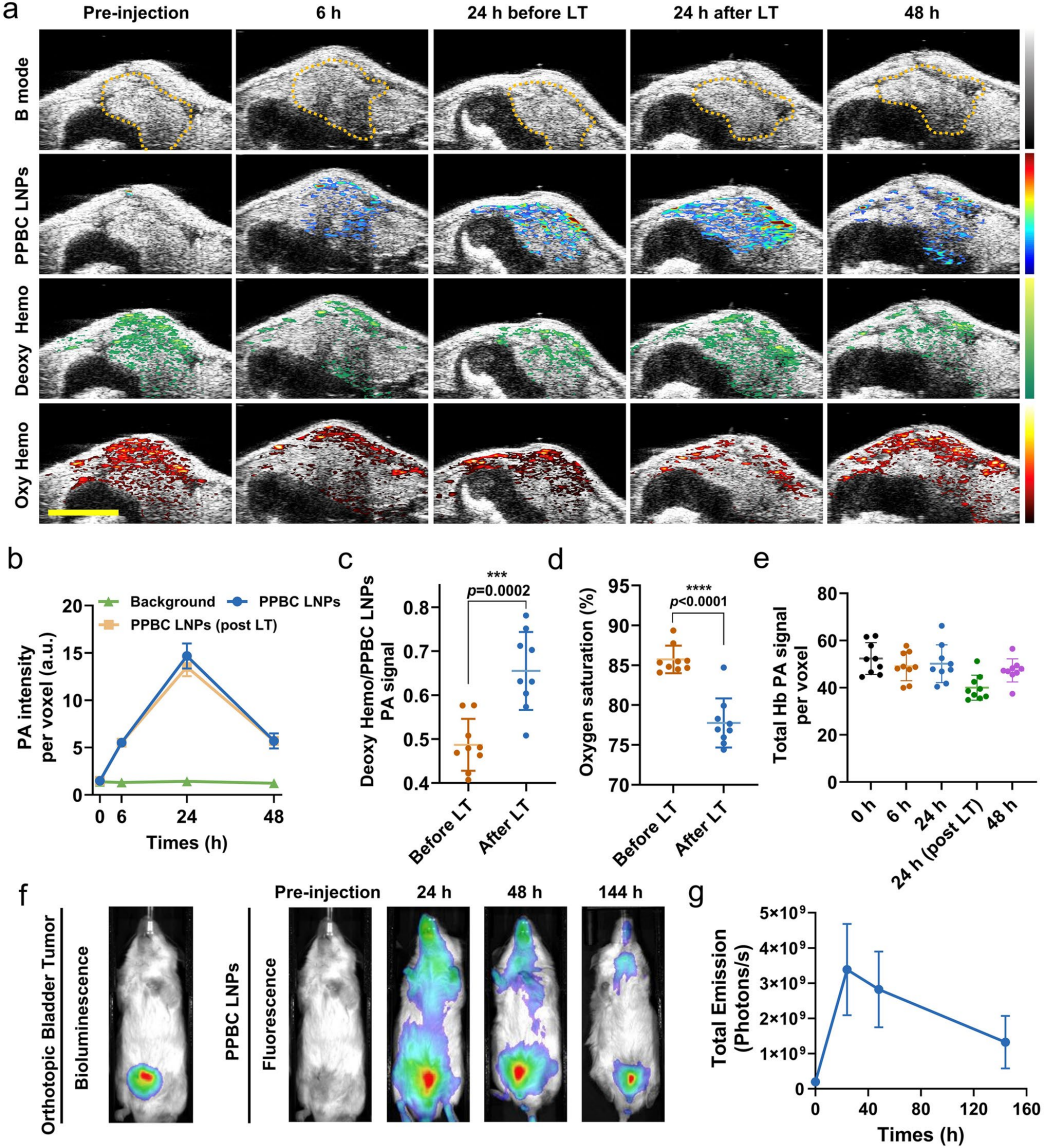
**a** In vivo PA signal patterns from PPBC LNPs, deoxyhemoglobin, and oxyhemoglobin were monitored overtime (*n* = 3); scale bar: 5 mm. LT: laser treatment. **b** Quantitative analysis of PA signals from PPBC LNPs within the tumor as shown in **a** (yellow dot-line circled). **c** Quantitative analysis of deoxyhemoglobin signal and **d** oxygen saturation within the tumor at 24 h before and after LT shown in **a**. LT: Laser treatment. **e** Quantitative analysis of PA signals from total hemoglobin within the tumor as shown in **a**. **f** Bioluminescence of orthotopic T24 tumors and fluorescence biodistribution of PPBC LNPs (i.v. 10 mg kg^−1^) in mice at various time points (*n* = 3). **g** Quantitative fluorescence signal analysis at tumor site shown in **f**

We next demonstrated the PA and FL imaging capabilities of PPBC LNPs in an orthotopic bladder tumor bearing mouse. First, US/PA imaging was performed both before and after the administration of PPBC LNPs via tail vein injection (10 mg kg^−1^). US/PA imaging is particularly advantageous in this orthotopic model, as ultrasound provides detailed anatomical context, while PA imaging detects signals from the contrast agent, PPBC LNPs. Additionally, by sweeping across NIR wavelengths, this approach allows for the simultaneous detection of PA signals from PPBC LNPs and endogenous signals from hemoglobin, differentiating between its oxygenated and deoxygenated status. By spectrally unmixing the different PA signal patterns from PPBC LNPs, deoxyhemoglobin, and oxyhemoglobin, we monitored nanoparticle accumulation and assessed key functional markers within the TME, such as oxygen saturation, which are crucial for PDT. This was achieved within the anatomical context provided by US imaging, using the same mouse over time (Fig. 4a). Pre-injection US/PA images helped identify the tumor mass within the bladder region from the B-mode ultrasound and both deoxygenated and oxygenated hemoglobin, yet no PPBC LNP signals presenting in images. After tail vein injection of PPBC LNPs, gradual increase in signal up to 24 h was monitored, and then, the signal decreased at 48 h, showing the kinetic accumulation of PPBC LNPs within the tumor mass (Fig. 4b). Notably, there was a significant change in blood signals (deoxyhemoglobin and oxyhemoglobin) following laser treatment, as observed through US/PA imaging. Specifically, the deoxyhemoglobin signal increased substantially in regions where PPBC LNPs had accumulated, suggesting oxygen consumption, primarily from the blood, for effective PDT (Figs. 4c and S11a). Furthermore, the quantitative measurements indicate that total hemoglobin levels remain largely unchanged after PPBC LNP injection (Fig. 4e), yet the light treatment significantly reduces overall oxygen saturation (Figs. 4d and S11b). This further indicates that ROS generation occurs primarily through the consumption of oxygen from hemoglobin, rather than from shifts in the total hemoglobin content in the tumor microenvironment. The application of this real-time, noninvasive functional US/PA imaging technique enhances our understanding of the role of hypoxia and metabolic factors in driving tumor progression and modulating therapeutic responses.

Simultaneously, biodistribution of PPBC LNPs was monitored via FL imaging using Lago X imaging system, with tumors tracked by the bioluminescence from luciferase-transfected T24 cells. As shown in Fig. 4f, g, FL imaging indicated that PPBC LNPs exhibited peak accumulation at the tumor site 24 h post-injection, which is consistent with PA imaging results. Remarkably, FL signals persisted for up to 6 days, showing the high sensitivity of FL imaging and, importantly, demonstrating the prolonged retention of PPBC LNPs in the tumor. This extended retention is particularly important, as it suggests the potential for sustained therapeutic effects through autophagy inhibition. Additionally, when combined with laser treatment, the prolonged post-injection window for phototherapy offers greater flexibility in treatment schedule, potentially improving patient compliance and convenience [10]. Ex vivo FL imaging of major organs and tumors revealed a substantial PPBC LNPs accumulation in tumors after 24 h i.v. injection, showing enhanced tumor accumulation compared to other reported PPA-based NPs (Fig. S12) [47]. Notable signals were also detected in the lung, liver, and kidney, which could be attributed to the approximately 100 nm size of the PPBC LNPs, potentially promoting lung accumulation and the renal clearance pattern of porphyrin derivatives, respectively [48, 49].

### In Vivo Evaluation of Phototherapeutic Effects

**Fig. 5:**
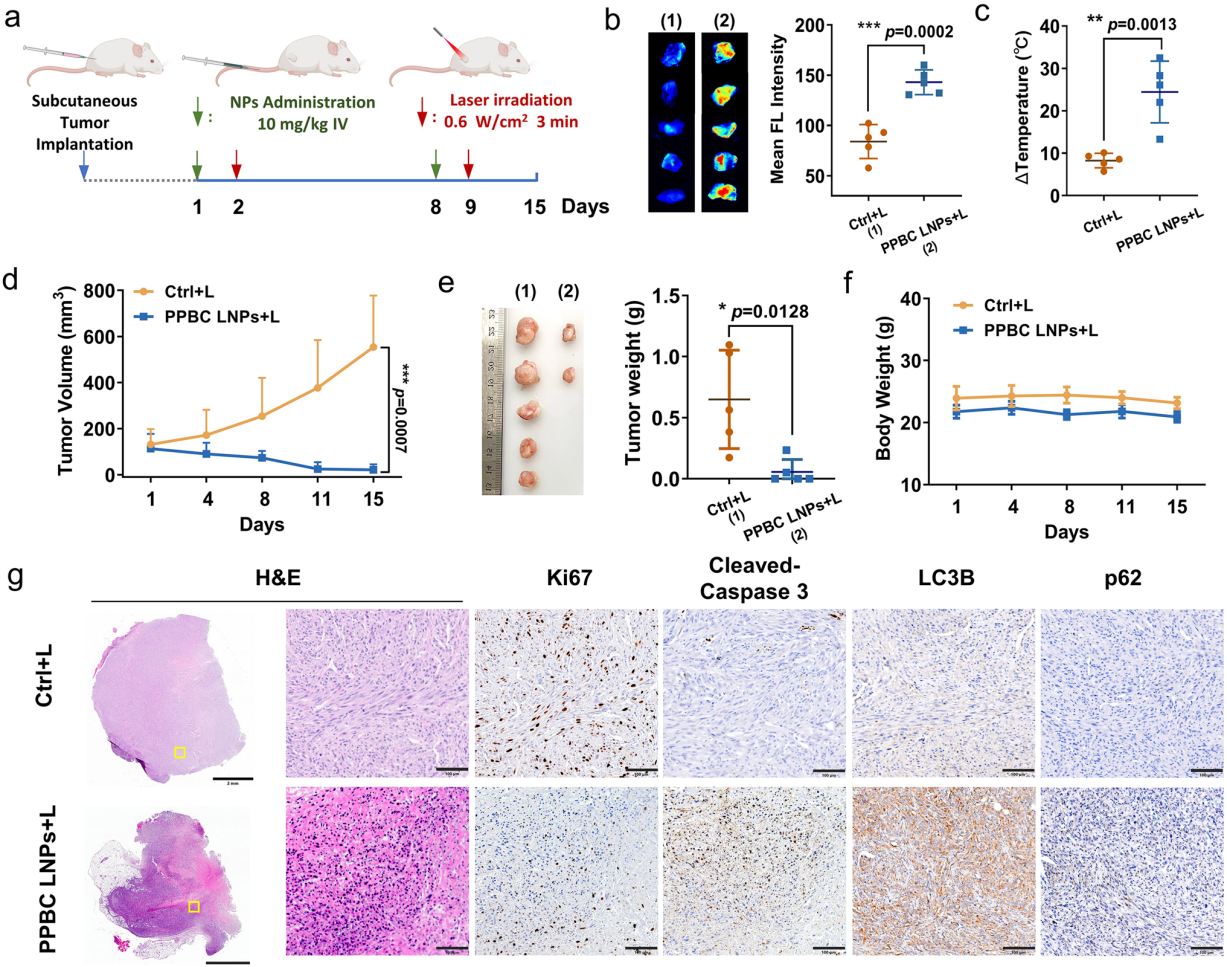
**a** Treatment schedules for subcutaneous bladder tumor (UPPL) models. PPBC LNPs (i.v. 10 mg kg^−1^) were injected on day 1 and 8. Tumors were then treated with laser (680 nm, 0.6 W cm^−2^, 3 min) 24 post-injection on each occasion (*n* = 5). **b** Ex vivo imaging and quantitative analysis of ROS production at the tumor site were performed after light irradiation using DCF-DA as the ROS indicator (*n* = 5). **c** Photothermal effect of PPBC LNPs illuminated by laser at the tumor site (*n* = 5). **d** Tumor volume change during treatment (*n* = 5). **e** Tumor weight at the end of treatment. **f** Body weight changes during treatment period. **g** H&E staining and IHC-Ki67, cleaved caspase-3, LC3B and p62 staining of subcutaneous tumor tissues, H&E: Hematoxylin and Eosin; IHC: Immunohistochemistry, scale bar: 2 mm (4 ×); 0.1 mm (20 ×)

To evaluate the photosensitization effect at tumor site, we first analyzed ROS and heat production upon PPBC LNPs-mediated phototherapy. Quantitative analysis based on fluorescence imaging showed significantly elevated ROS levels in the tumor (Fig. 5b). In addition, PPBC LNPs demonstrated a strong photothermal effect, significantly increasing the temperature at the tumor site (Fig. 5c). These results indicate that PPBC LNPs has substantial potential for in vivo phototherapy.

Next, we assessed the antitumor efficacy of PPBC LNPs-mediated phototherapy. Mice bearing subcutaneous bladder tumors received PPBC LNPs (i.v. 10 mg kg^−1^) on days 1 and 8. Based on PA and FL imaging results, PPBC LNPs showed a peak accumulation at the tumor site 24 h post-injection. Therefore, tumors were exposed to laser irradiation (680 nm, 0.6 W cm^−2^) for 3 min at 24 h after each administration (Fig. 5a). As shown in Fig. 5d, e, compared to the control group, the growth of bladder tumors treated with PPBC LNPs and laser was significantly inhibited, with three out of five tumors completely ablated after just two treatment cycles, demonstrating the potent effects of PPBC LNPs-mediated phototherapy. Furthermore, the treatment showed good safety, as evidenced by stable body weights throughout the treatment period (Fig. 5f). To further investigate tissue-level changes, tumors were collected at the end of treatment, and the expression of specific proteins was analyzed. As depicted in Fig. 5g, compared to the control group, tumors treated with PPBC LNPs and laser showed significant tissue damage (H&E), reduced Ki-67 (cell proliferation marker) expression, and increased cleaved caspase-3 (apoptosis marker), LC3B and p62 levels (autophagy inhibition markers), suggesting decreased proliferation, enhanced apoptosis and autophagy suppression at the tumor site. Histopathological evaluation of the major organs showed no abnormalities, further confirming the biocompatibility of the treatment (Fig. S13). These results demonstrate that PPBC LNPs can effectively suppress and even ablate tumors by inhibiting autophagy and inducing apoptosis, highlighting their potent autophagy inhibition and phototherapeutic effects in vivo.

**Fig. 6:**
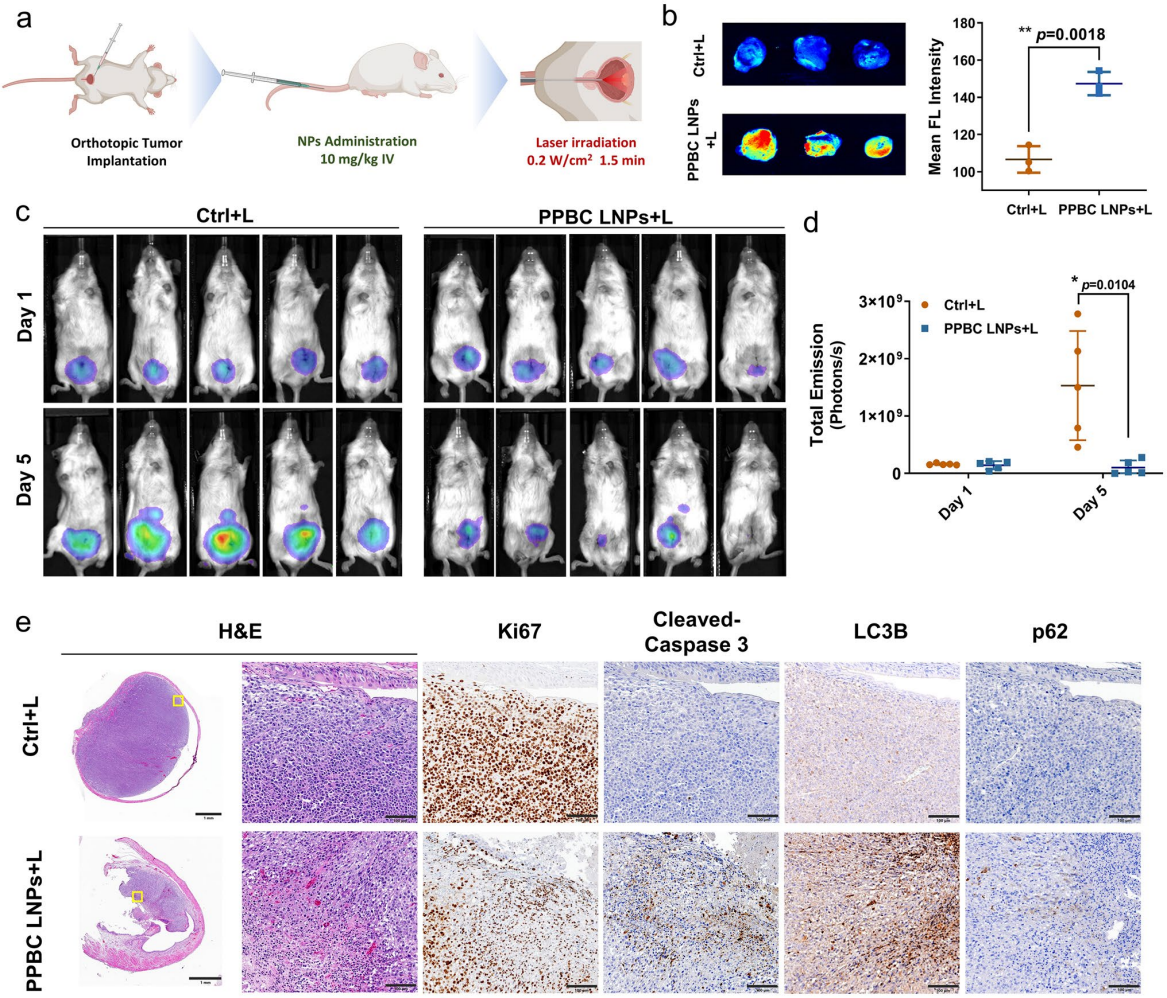
**a** Treatment illustration for orthotopic bladder tumor (T24) models. PPBC LNPs (10 mg kg^−1^) were i.v. injected intravenously on day 1. Tumors were then treated with laser (680 nm, 0.2 W cm^−2^, 1.5 min) 24 and 48 h post-injection. **b** Ex vivo imaging and quantitative analysis of ROS production at the tumor site were performed after light irradiation (24 h post-injection) using DCF-DA as the ROS indicator (*n* = 3). **c** Representative bioluminescence images and **d** quantitative data of tumor region from mice that treated with or without PPBC LNPs for 24 h, followed with laser treatment (*n* = 5). **e** H&E staining and IHC-Ki67, cleaved caspase-3, LC3B and p62 staining of orthotopic tumor tissues, H&E: Hematoxylin and Eosin; IHC: Immunohistochemistry, scale bar: 1 mm (4 ×); 0.1 mm (20 ×)

To better evaluate the therapeutic effects of PPBC LNPs in bladder cancer, we used an orthotopic bladder cancer mouse model and the whole bladder was illuminated with a 400-micron optical fiber (Fig. 6a). Consistent with data in Fig. 5, PPBC could specifically accumulate at orthotopic bladder cancer sites, and significantly increase ROS production upon PPBC LNP-mediated phototherapy was observed (Fig. 6b).

The mice bearing orthotopic bladder tumors were divided into two groups and treated with or without PPBC LNPs (i.v. 10 mg kg^−1^), followed by laser treatment 24 h after administration. On day 5, tumor growth was monitored by measuring luciferase activity. Bioluminescence imaging showed a dramatic increase in tumor signals in the control group, indicating aggressive growth of the orthotopic bladder tumors (Fig. 6c). In contrast, the group treated with PPBC LNPs plus laser showed significantly reduced bioluminescent signals (Fig. 6d). Notably, tumor signal was completely absent in one mouse, suggesting complete tumor ablation following treatment. Similar to xenograft model, histological examination revealed obvious necrosis and fragmentation of orthotopic bladder cancers. IHC analysis confirmed that PPBC LNP-mediated light treatment caused a decreased cell proliferation (reduced Ki67), increased apoptosis (elevated cleaved caspase 3), and autophagy inhibition (increased LC3b and p62) in bladder tumor tissues (Fig. 6e). No obvious toxicity was noted in major organs (Fig. S14).

In this study, we developed pyropheophorbide a–bisaminoquinoline conjugate lipid nanoparticles (PPBC LNPs) designed to integrate trimodal therapeutic functions (PDT, PTT, and autophagy inhibition) and bimodal imaging capabilities (FL and PA imaging) into a single platform. In comparison with the studies performed previously using similar platforms, this study holds great promise for lab-to-clinical translation through two key features. First, the well-defined structure of PPBC eliminates complications related to stereoisomerism and structural variability, ensuring consistent chemical composition and therapeutic efficacy across batches, which is critical for regulatory approval process. Second, employing microfluidics-assisted formulation offers precise control over particle size and composition, ensuring uniformity across batches [50]. Furthermore, this method is inherently scalable, allowing large quantities without compromising quality, a key factor in meeting clinical demand. In the current study, PPBC LNPs demonstrated efficient tumor accumulation and significant inhibition of tumor growth when combined with laser irradiation, resulting in the complete ablation of several tumors in bladder cancer models. These findings highlight the potential of PPBC LNPs as a multifunctional platform for bladder cancer therapy, warranting further investigation and development toward clinical application.

The multifunctional “all-in-one” design of PPBC LNPs offers distinct advantages in the treatment of bladder cancer. Traditional PSs, such as Hypericin and Photofrin, are limited by the short half-life and restricted diffusion of ^1^O_2_, and oxygen depletion during therapy, which reduces their efficacy [51, 52]. Furthermore, conventional PDT often triggers autophagy, which can act as a survival mechanism for cancer cells, potentially leading to resistance [53, 54]. In bladder cancer, autophagy activation has been linked to resistance against radiotherapy and chemotherapies such as gemcitabine [55, 56]. PPBC LNPs overcome these limitations by integrating strong PDT and PTT effects with effective autophagy inhibition. As demonstrated in Figs. 5 and 6, PPBC LNPs achieved complete tumor ablation in bladder cancer models after just one or two treatment cycles. In comparison, Ce6- or hematoporphyrin-based PDT or PTT requires more treatment cycles or longer laser irradiation to achieve similar effects in bladder cancer, increasing the risk of undesirable side effects [57, 58]. Given the differences in bladder sizes and treatment windows between murine models and humans, further efficacy investigation of PPBC LNPs in larger animal models is necessary and is currently underway [13].

Moreover, PPBC LNPs integrate PA and FL imaging capabilities directly within the therapeutic agent, eliminating the need for additional imaging dyes. This integration offers several advantages: It simplifies the overall nanoparticle formulation, reduces potential toxicity from added agents, and ensures consistent pharmacokinetics. Compared to other nanomedicines for bladder cancer currently in clinical trials, such as EP-0057 and Imx110 [59], which often involve complex synthesis processes with multiple components, PPBC LNPs offer a more efficient and practical approach for large-scale production. The well-defined small-molecule structure serves as the photosensitizer, autophagy inhibitor, imaging agent, and nanostructure component, simplifying and streamlining the synthesis and formulation process and reducing production costs and time.

Although PA or combined US/PA imaging offers high-resolution images with greater penetration depth compared to many optical modalities, translating these advantages into clinical applications remains challenging due to the attenuation of laser light in tissue [23, 60]. While our approach achieved sufficient imaging depth for detecting bladder tumors in a small animal model, the deeper location of the human bladder necessitates the development of more advanced strategies to enable robust clinical imaging [61, 62]. One promising avenue involves the use of internal-illumination photoacoustic techniques and photoacoustic endoscopy. These methods, which utilize optical fibers or integrated transducers introduced endoscopically or via catheters, enable imaging at depths beyond the limitations of conventional external-illumination PA systems, facilitating detailed visualization of internal organs such as the bladder [63–65]. These systems have also demonstrated their clinical utility in applications such as gastrointestinal tract imaging, prostate cancer diagnosis, and beyond [66–68]. Future efforts could focus on integrating catheter-based probes or endoscopic approaches into our PA imaging platform, thereby addressing current limitations and enhancing the translational potential of this technology for bladder cancer diagnosis and monitoring therapeutic responses.

## Conclusion

In conclusion, the unique design of PPBC LNPs as a self-sufficient nanoplatform, with its potent phototherapy effects, autophagy inhibition properties, and bimodal imaging capabilities, holds great promise for bladder cancer therapy. The scalability of their production and their multifunctional therapeutic and imaging capabilities support their potential for clinical development, warranting further investigation in larger animal models, which is part of our upcoming studies, and eventual translation to human patients.

## Supplementary Information

Below is the link to the electronic supplementary material.

Supplementary file1 (DOCX 6332 KB)
