# Supplementary material for: Multifunctional and Scalable Nanoparticles for Bimodal Image-Guided Phototherapy in Bladder Cancer Treatment
Source: Nanomicro Lett. 2025 Apr 18;17:222. doi: 10.1007/s40820-025-01717-0 (PMC12008111; doi:10.1007/s40820-025-01717-0)
Supplement: Supplementary file 1 — Supplementary file1 (DOCX 6332 KB) [file 40820_2025_1717_MOESM1_ESM.docx]

# Supporting Information for

**Multifunctional and Scalable Nanoparticles for Bimodal Image-Guided Phototherapy in Bladder Cancer Treatment**

Menghuan Tang^1^, Sohaib Mahri^1^, Ya-Ping Shiau^1^, Tasneem Mukarrama^2, 3^, Rodolfo Villa^1^, Qiufang Zong^1^, Kelsey Jane Racacho^1^, Yangxiong Li^1^, Yunyoung Lee^2, 3^, Yanyu Huang^1^, Zhaoqing Cong^1^, Jinhwan Kim^2, 3,^ *, Yuanpei Li^1^ *, Tzu-Yin Lin^4^ *

^1^ Department of Biochemistry and Molecular Medicine, UC Davis Comprehensive Cancer Center, University of California Davis, Sacramento, CA 95817, USA

^2^ Department of Biomedical Engineering, University of California, Davis, Davis, CA 95616, USA

^3^ Department of Surgery, School of Medicine, University of California, Davis, Sacramento, CA 95817, USA

^4^ Division of Hematology/Oncology, Department of Internal Medicine, University of California Davis, Sacramento, CA 95817, USA

*Corresponding authors. E-mail: [jjnkim@ucdavis.edu](mailto:jjnkim@ucdavis.edu) (Jinhwan Kim); [lypli@ucdavis.edu](mailto:lypli@ucdavis.edu) (Yuanpei Li); [tylin@ucdavis.edu](mailto:tylin@ucdavis.edu) (Tzu-Yin Lin)

**Supplementary Figures**


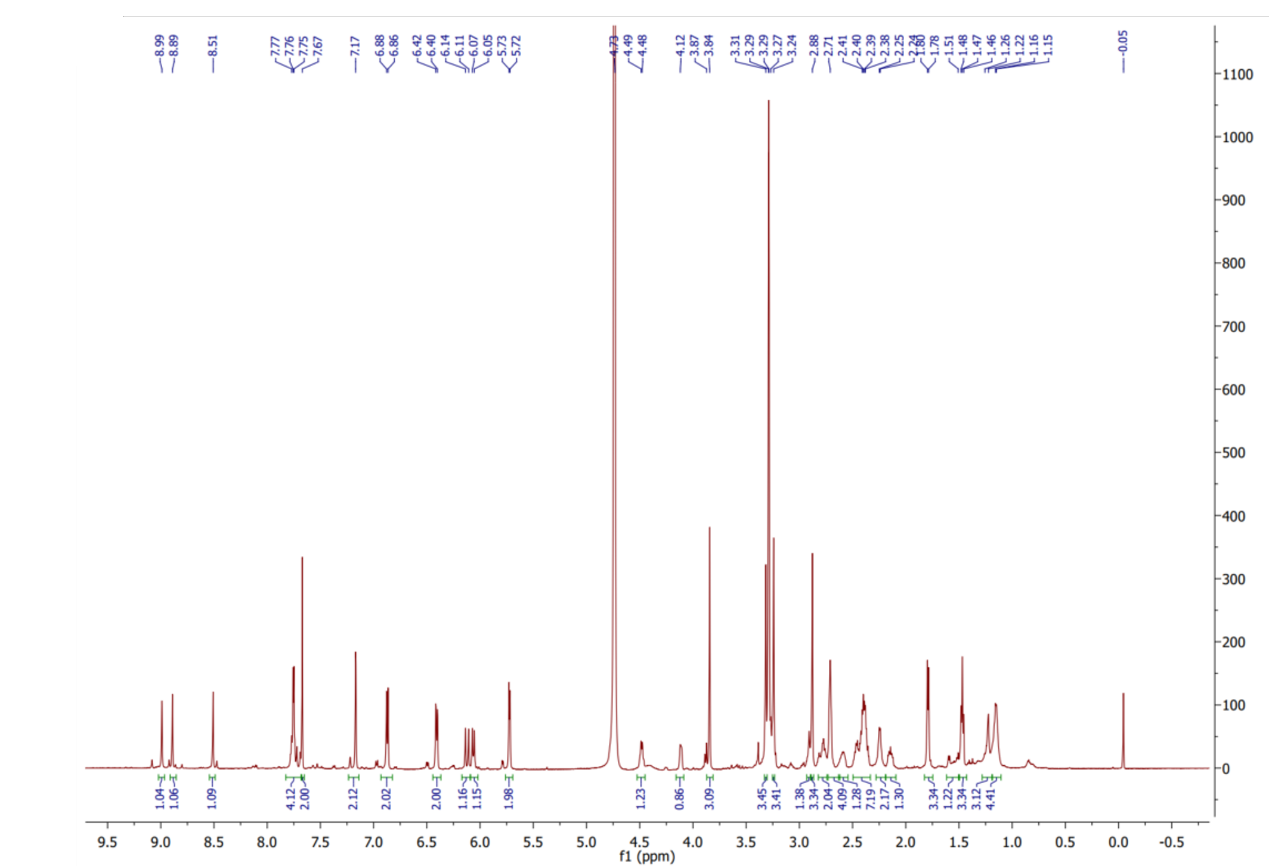


**Fig. S1** ^1^H NMR (600 MHz, MeOD) of PPBC


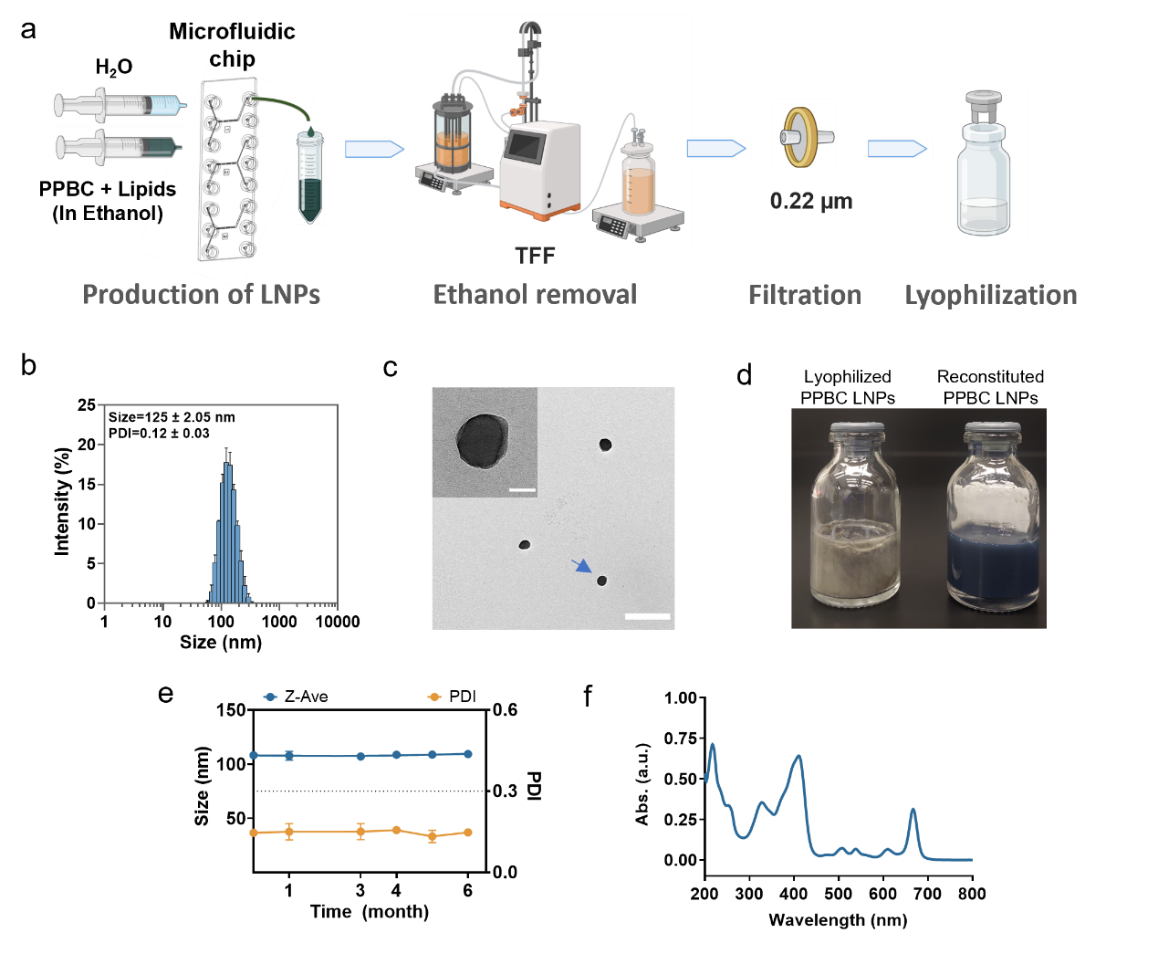


**Fig. S2** **a**) Workflow illustrating the use of microfluidics for scalable production of PPBC LNPs, TFF: tangential flow filtration. **b**) Representative DLS data and **c**) TEM image of reconstituted PPBC LNPs, scale bar: 50 nm (insert), 400 nm. **d**) The pictures of lyophilized and reconstituted PPBC LNPs. **e**) Stability study of lyophilized PPBC LNPs. **f**) UV-vis spectrum of PPBC


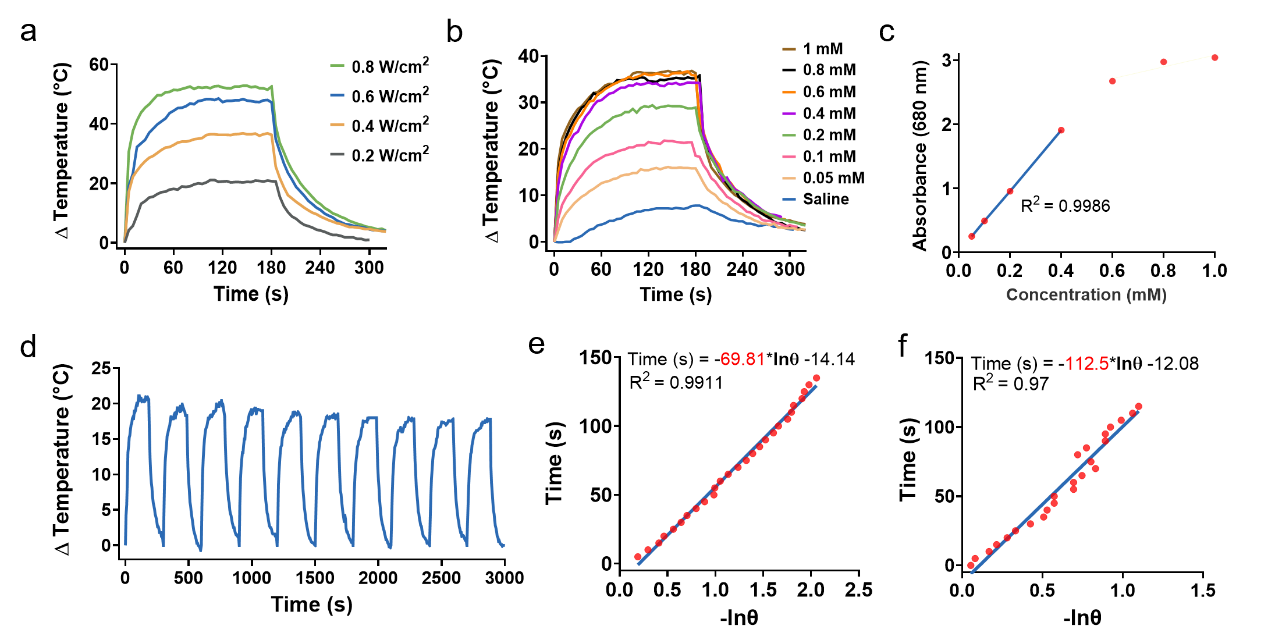


**Fig. S3** **a**) Temperature curves for a solution of 0.4 mM PPBC LNPs at different laser intensities. **b**) Temperature curve of different concentrations of PPBC LNPs or saline at 0.4 W/cm^2^ laser intensity. **c**) Calibration curve of PPBC LNPs at 680 nm and fitting of the linear range (0.05 mM to 0.4 mM). **d**) Photothermal stability of PPBC LNPs at 0.1 mM using laser intensity of 0.4 W/cm^2^ for 10 heating-cooling cycles, each cycle consisting of 3 min irradiation following by 2 min cooling. **e**) Linear fitting between time (t) and -lnθ (θ = ΔT/T_max_) from the cooling period of PPBC LNPs solution after 3 min laser irradiation at 0.4 W/cm^2^. **f**) Linear fitting between time (t) and -lnθ (θ = ΔT/T_max_) from the cooling period of saline after 3 min laser irradiation at 0.4 W/cm^2^

**Fig. S4** The cellular uptake of PPBC LNPs at different concentrations by T24 cells. Data are present as means ± SD (n=3)

**Fig. S5** Dose-dependent inhibition of Genistein on cellular uptake of PPBC LNPs by T24 cells. Values are mean ± SD (n=3)


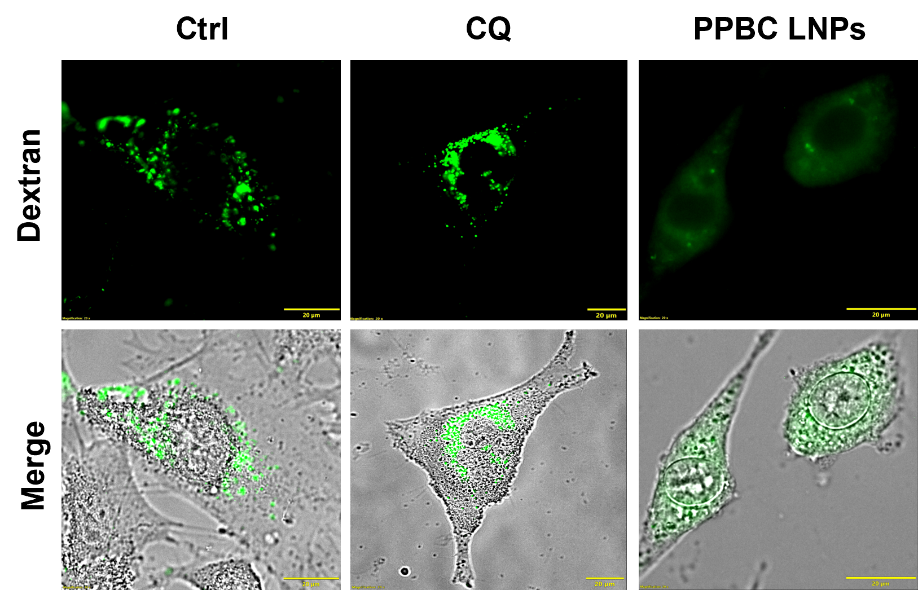


**Fig. S6** Representative images of Alexa Fluor 488-Dextran-loaded UPPL cells were exposed to chloroquine (CQ,10 μM) or PPBC LNPs (10 μM) for 24 h, scale bar: 20 μm


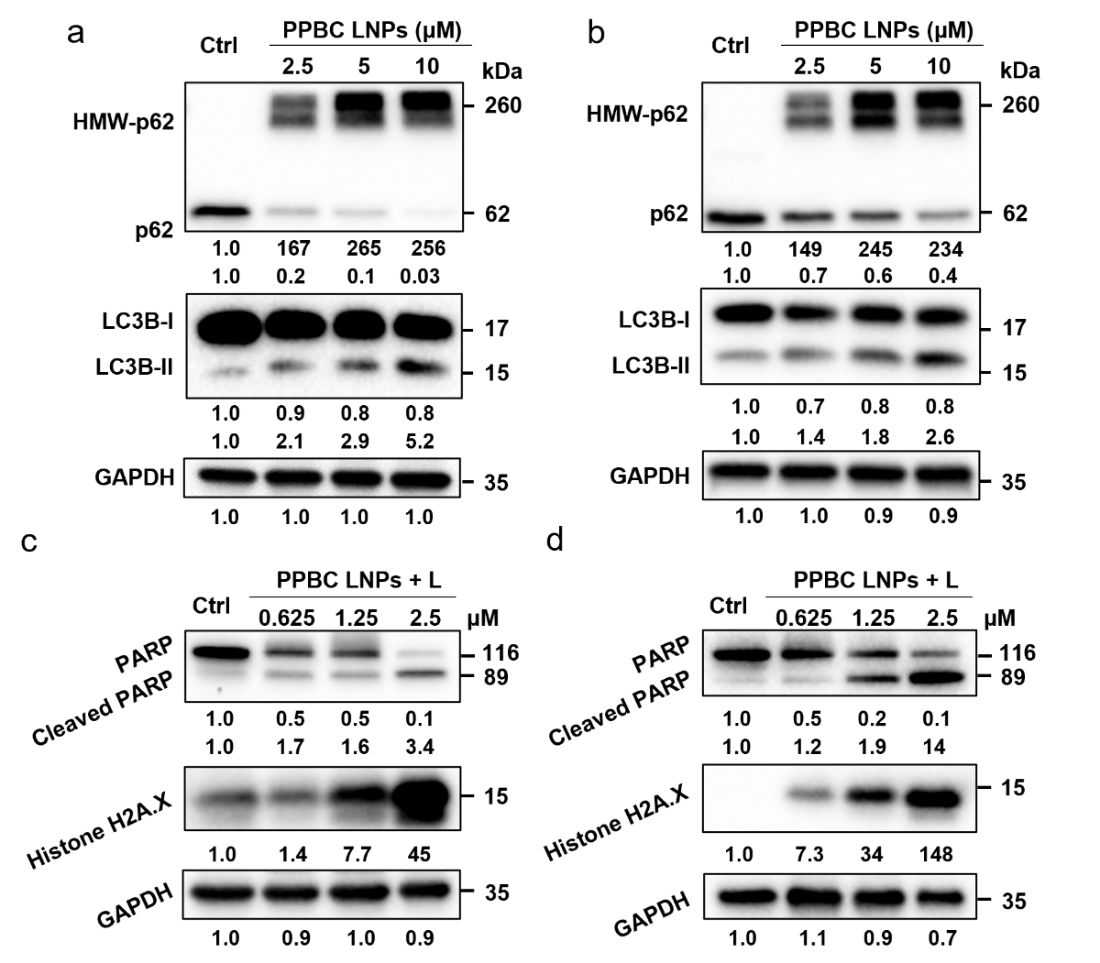


**Fig. S7** Immunoblotting and relative intensity analysis of autophagy-related proteins (p62, LC3B) in (**a**) T24 and (**b**) UPPL cells treated as indicated for 12 h. Immunoblotting and relative intensity analysis of apoptosis and DNA damage-related protein (PARP, cleaved-PARP, Histone H2A.X) in (**c**) T24 and (**d**) UPPL cells treated with PPBC LNPs plus light. L: with light irradiation, 30 mW/cm^2^ (633 nm LED array) for 30 s


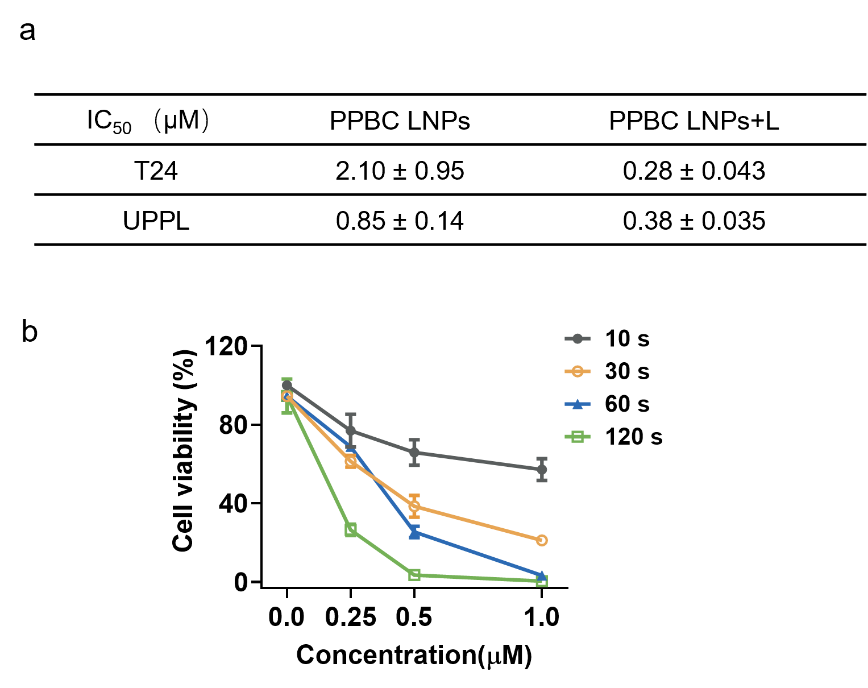


**Fig. S8** (**a**) IC_50_ values of cytotoxicity. (L: with light irradiation. Light treatment: 30 mW/cm^2^ (633 nm LED array) for 30 s. (**b**) Viability curves of UPPL expose to different light treatment duration. Light treatment: 30 mW/cm^2^ (633 nm LED array). Values are mean ± SD (n=3)

**
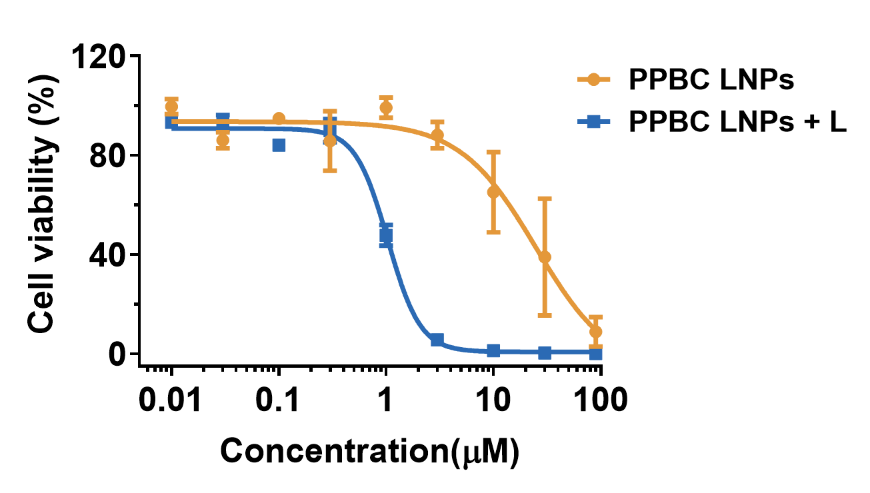
**

**Fig. S9** Viability curves of IMR90 cells exposed to PPBC LNPs with or without light treatment. L: with light irradiation. Light treatment: 30 mW/cm^2^ (633 nm LED array) for 30 s. Values are mean ± SD (n=3)


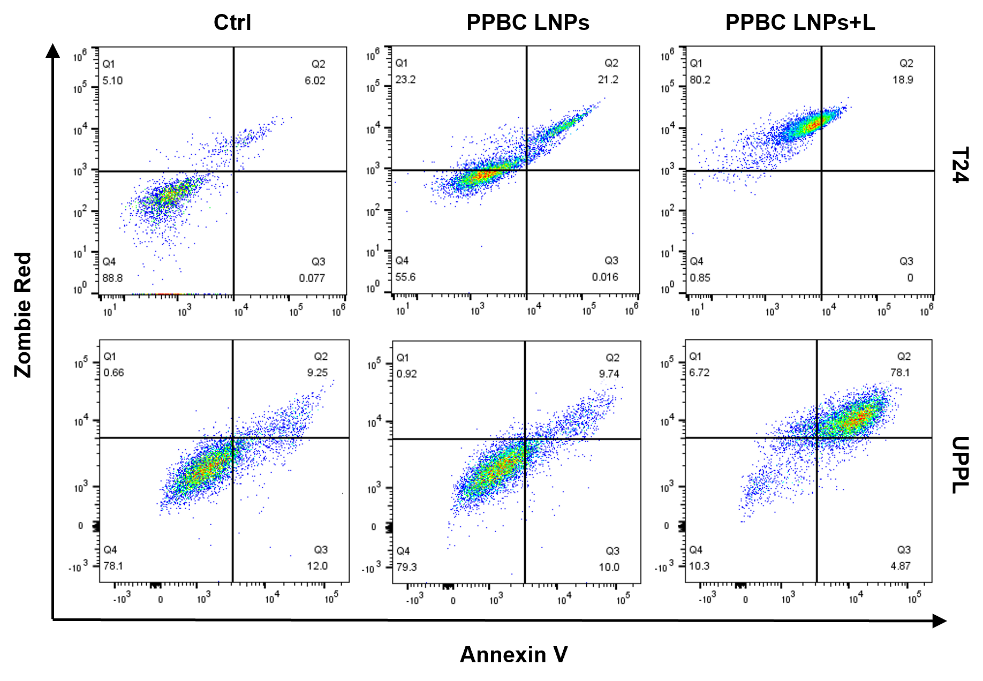


**Fig. S10** Apoptosis assay of T24 and UPPL that were treated with 0.5 μM of PPBC LNPs for 24 h, followed by light treatment (30 mW/cm^2^, 5 min) and another incubation for 24 h

**
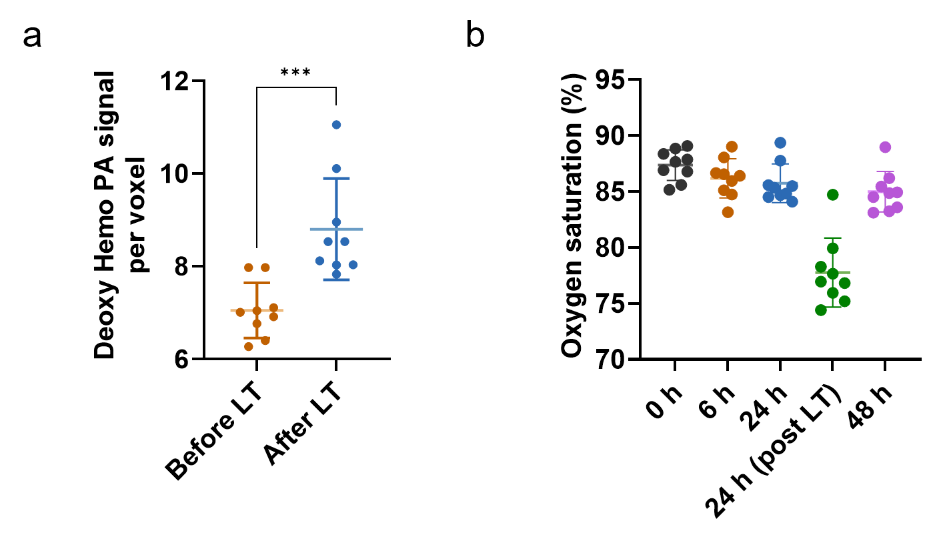
**

**Fig.** **S11** Quantitative analysis of PA signals from (**a**) deoxyhemoglobin signal and (**b**) oxygen saturation within the tumor as shown in **Fig.** 4(a)

_
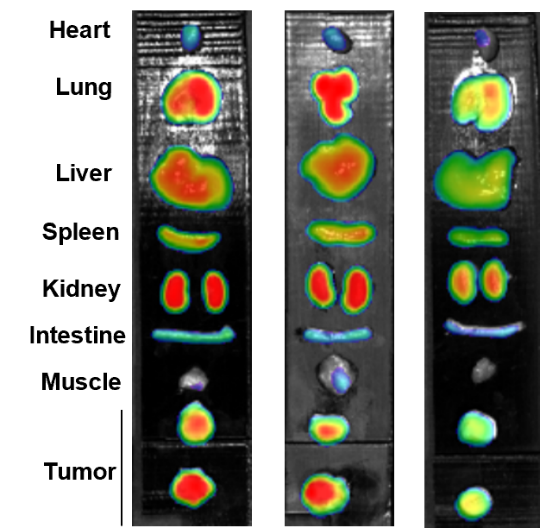
_

**Fig. S12** *Ex vivo* imaging of the heart, lung, liver, spleen, kidney, intestine, muscle, and tumor tissue (The bladder tumor was halved to better visualize drug accumulation within the tumor) from orthotopic bladder cancer models performed at 24 hours post-injection

_
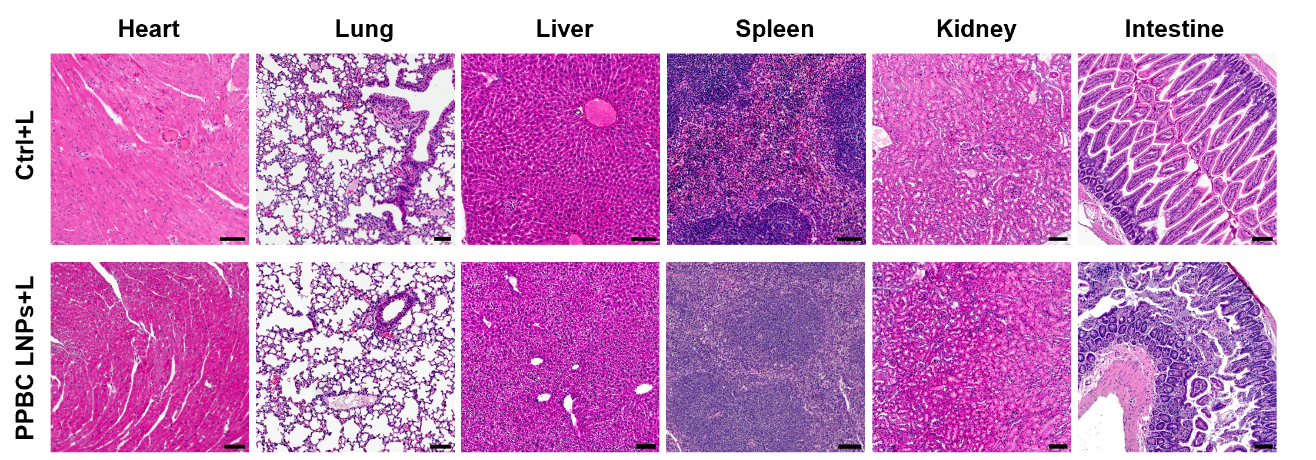
_

**Fig. S13** H&E staining of main organs from mice bearing subcutaneous bladder tumor to evaluate systemic toxicity following treatment; scale bar: 100 μm

_
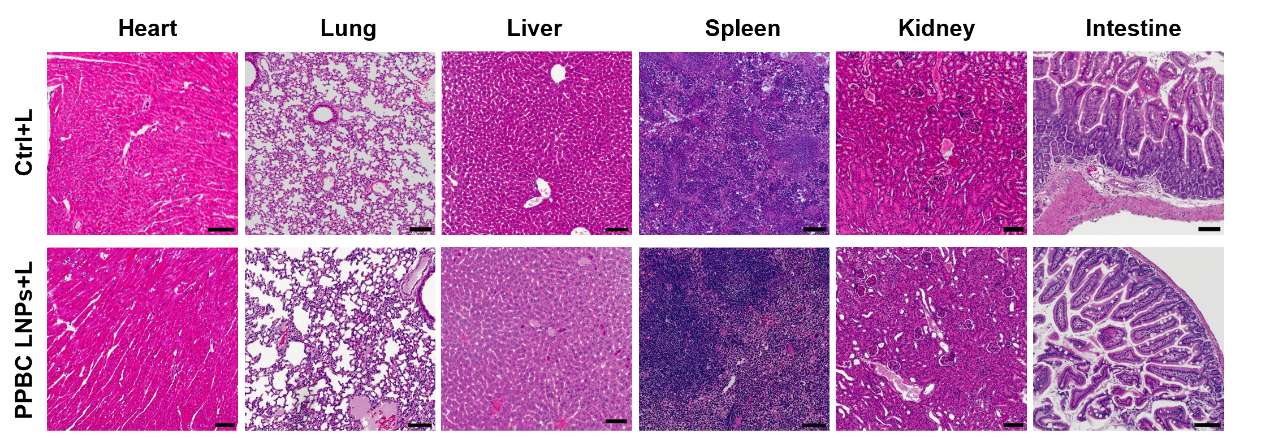
_

**Fig. S14** H&E staining of main organs from mice bearing orthotopic bladder tumor to evaluate systemic toxicity following treatment; scale bar: 100 μm
